# Computer simulation and physical phantom models for estimating the dielectric properties of rhinoceros tissue

**DOI:** 10.1371/journal.pone.0216595

**Published:** 2019-05-29

**Authors:** Floris J. van Zyl, Johan Marais, Martin Nieuwoudt, Thomas R. Niesler

**Affiliations:** 1 Department of Electrical and Electronic Engineering, Stellenbosch University, Stellenbosch, Western Cape, South Africa; 2 Faculty of Veterinary Science, University of Pretoria, Pretoria, Gauteng, South Africa; 3 Institute for Biomedical Engineering (IBE), Stellenbosch University, Stellenbosch, Western Cape, South Africa; Cranfield University, UNITED KINGDOM

## Abstract

*In vivo* and *ex vivo* sensors have the potential to aid tracking and anti-poaching endeavours and provide new insights into rhinoceros physiology and environment. However, the propagation of electromagnetic signals in rhinoceros tissue is currently not known. We present simulation and agar models of the rhinoceros that allow the investigation of electromagnetic propagation by *in vivo* and *ex vivo* devices without the need for surgery. Since the dielectric properties of rhinoceros tissue have not been documented, the conductivity and permittivity of the skin, fat, muscle, blood and other organs are first approximated by means of a meta-analysis that includes animals with similar physical properties. Subsequently, we develop anatomical models that include dermal layers, internal organs and a skeleton. We also develop a flank model that serves as an approximation of the anatomical model in certain situations. These models are used to determine the viability of communication between an *in vivo* device and an *ex vivo* device attached to the hind leg of the animal. Two types of antenna (microstrip-fed planar elliptical monopole antenna and printed inverted-F antenna) and three feasible implant locations (back, neck and chest) are considered. In addition to the computer models, phantom recipes using salt, sugar and agar are developed to match the dielectric properties of each tissue type at the industrial, scientific and medical (ISM) frequencies of 403MHz, 910MHz and 2.4GHz. The average error between the measured and theoretically predicted dielectric values was 6.22% over all recipes and 4.49% for the 2.4 GHz recipe specifically. When considering the predicted efficiency of the transmitting and receiving antennas, an agreement of 67.38% was demonstrated between the computer simulations and laboratory measurements using the agar rhinoceros flank models. Computer simulations using the anatomical model of the rhinoceros indicate that the chest is the optimal implant location and that best signal propagation is achieved using the planar inverted-F antenna (PIFA). Using this configuration, the simulations indicate that communication between the implant and an *ex vivo* device attached to the hind leg is challenging but possible. Furthermore, we find that the inclusion of factors such as the density and temperature of the phantom materials were found to be critical to the achievement of good agreement between practice and simulation.

## Introduction

Although rhinoceroses have few natural predators, the African black rhinoceros (*Diceros bicornis*) and the African white rhinoceros (*Ceratotherium simum*) are nearing extinction in the wild due to active poaching by humans. The prevention of this illegal practice is impeded by the vastness of the habitat. Attempts to monitor and study these animals using *ex vivo* attached devices are made difficult by the anatomical structure of the rhinoceros, their regular exposure to confrontational impact during intraspecific territorial combat and the difficulty of achieving wireless communication with such devices. Furthermore, it is not possible to reliably measure most physiological signals, such as heartrate, using *ex vivo* sensors due to the animal’s thick hide and the hostile environment.

Little is known about the physiological, social and migratory behaviour of the rhinoceros. This inhibits the optimization of devices specifically designed for monitoring these animals in the wild. To design better *ex vivo*, as well as new *in vivo* wireless sensors, the electromagnetic properties of the rhinoceros body must be, at least approximately, known. Active radio frequency implantation and animal telemetry devices typically communicate using frequencies ranging from the low kilohertz region (30 kHz) to the higher megahertz region (915 MHz). The lower frequencies are often preferred in wildlife telemetry devices due to their longer range. However, due to the abnormally thick skin of the rhinoceros, higher frequencies may be more appropriate owing to their superior penetrative ability. Currently, the only direct means of comparing alternative communication strategies is live field testing, using either external attachments or implantation by means of surgery. This is practically difficult and potentially dangerous for both the researchers and the rhinoceros.

Computer simulation and physical phantom models which replicate the dielectric properties of the rhinoceros could be used to aid the design and development of new animal-attached and implanted sensors to support anti-poaching initiatives. Such models would provide a test environment for experimental *in vivo* and *ex vivo* devices used for tracking and monitoring animal movement and physiology [1, 2]. The intended purpose of such implantation devices is to transmit data through the thick hide of a rhinoceros. Thus, the models should sufficiently portray the radiation attenuation of rhinoceros tissue.

In this paper, we design computer simulation models with different degrees of complexity as well as physical phantom models for the purpose of enabling the design of communication strategies for *in vivo* and *ex vivo* sensors for the rhinoceros. All considered frequencies are within the Industrial, Scientific and Medical (ISM) bands. As a specific goal, the developed models were used to consider communication between an *in vivo* transmitter and *ex vivo* receiver in order to investigate viable antenna designs and possible points of implantation. Both the computational and the physical models proposed in this paper are to our knowledge currently the only approximations of rhinoceros tissue of their kind.

The structure of the remainder of this paper is as follows. Section 2 elaborates on the rhinoceros tissue approximation methods and the materials used to develop physical and numerical simulation models. This includes an estimation of the dielectric properties of rhinoceros tissue for various frequencies to allow the approximation and formulation of agar phantom material recipes. The estimated dielectric tissue properties and recipes were used to construct sample plates which were compared with the theoretical estimates. Section 3 describes the development and configuration of the numerical simulation models and elaborates on the results of the simulated power measurements. Section 4 provides a discussion of the results and Section 5 concludes the paper.

## Materials and methods

### Approximation of the dielectric properties of rhinoceros tissue

Little is known about the anatomy and dielectric properties of the rhinoceros. Yet this information is required to accurately develop simulation and phantom models that exhibit the expected signal mitigation characteristics. Since the dielectric properties of rhinoceros tissue and organs have yet to be measured using physical rhinoceros tissue samples, we have approximated these values by means of a meta-analysis using the permittivity and conductivity of animals with similar characteristics. Constituent animal contributions to a specific organ or tissue were calculated by applying a weighted average based on a decision matrix of various physical characteristics, which favoured animals with greater similarity to the rhinoceros. Each characteristic was assigned a level of importance that distinguished between essential and auxiliary identifiers for approximating the model. A multiplier system was used to ascribe greater weight to characteristics of higher importance.

The animals used for approximating rhinoceros tissue are listed in Table 1. This table portrays the results of the decision matrix which describes the percentage of each animal’s contribution to the rhinoceros model as a whole. The weighting factors were derived by considering individual physical attributes of these animals, such as the collagen content of their skin, the thickness of their subcutaneous fat layer and their bone structure. Our analysis indicates that equine, bovine, pinniped and porcine are most similar to rhinoceros and consequently have the greatest influence on the dielectric properties of the tissue. In particular, bovine were identified as having similar skeletal characteristics to rhinoceroses while equine have similar intestinal and organ characteristics. By applying the weighting factors depicted in Table 1 per tissue to the dielectric properties of animal tissue measurements found in the literature, the rhinoceros tissue dielectric approximations for the frequencies of 403 MHz, 910 MHz and 2.4 GHz were calculated, with the results indicated in Table 2. The physical attributes used to determine the comparability of the rhinoceros with various animals and the similarity between particular characterisitcs are indicated in S1 Table of the supporting information.

**Table 1:** Weighting factors of animals contributing to the approximation of rhinoceros tissue dialectric properties.

Weighting Factors												
Species	Equine	Bovine	Pinniped	Porcine	Ovine	Human	Rabbit	Canine	Feline	Rat	Mouse	Frog
**Weight**	0.1659	0.1647	0.1094	0.1075	0.1031	0.0951	0.0559	0.0534	0.0528	0.0434	0.0421	0.0069
**Contribution**	17%	16%	11%	11%	10%	10%	6%	5%	5%	4%	4%	1%

Contributions by different animal species to the approximation of rhinoceros tissue dielectric properties. These contributions are applicable to specific organs, as well as to the rhinoceros model as a whole.

**Table 2:** Tissue permittivity and conductivity approximations for rhinoceros tissue.

Biological Tissue	Approximated Permittivity			Approximated Conductivity		
	403 MHz	910 MHz	2.4 GHz	403 MHz	910 MHz	2.4 GHz
**Blood**	63.98	59.14	54.14	1.27	1.49	2.56
**Bone Cancellous**	19.21	17.50	15.78	0.19	0.34	0.68
**Bone Cortical**	10.89	9.78	8.78	0.10	0.15	0.29
**Fat**	11.36	10.07	8.29	0.09	0.11	0.18
**Grey Matter**	63.03	54.35	43.85	0.87	1.17	2.29
**White Matter**	47.80	36.44	32.53	0.51	0.82	1.44
**Kidney**	48.58	45.32	40.56	1.12	1.69	2.68
**Spleen**	55.01	49.49	45.83	1.08	1.50	2.12
**Heart**	45.17	41.26	34.47	1.14	1.61	2.47
**Liver**	49.49	43.46	39.14	0.90	1.14	1.75
**Lung**	35.65	31.91	27.57	0.55	0.75	1.05
**Muscle**	65.94	59.23	53.40	1.12	1.36	2.06
**Skin**	41.24	39.79	36.41	0.48	0.74	1.43

The results of applying the the weigting factors in Table 1 to determine the approximated dielectric properties of rhinoceros organs and tissue.

Three locations were considered for the implanted antenna—one in the neck of the rhinoceros above the shoulders, one below the head in the neck and one in the chest. Since *in vivo* antennas are typically embedded in the fat layer as a means to protect the implanted device, the dielectric properties of the thick skin of the rhinoceros is an important signal attenuation factor to consider. Thus, the chemical composition of rhinoceros skin was used to derive a second approximation for the permittivity of the rhinoceros dermis. By calculating the permittivity of the individual skin constituents, the collective permittivity of the skin was estimated. According to Shadwick et al [3], the dorsolateral and abdominal skin of the rhinoceros has a water content of approximately 60.9% (± 1.2%). The collagen content of the dry faction is 85% and the collagen content of the tissue wet mass is 33.2%. The dielectric constant of water was assumed to be 80 [4] while the dielectric constant of wet and dry collagen were assumed to be 4.5 and 2.3 respectively [5]. The remaining tissue was approximated as 0.1MNaCl, which has dielectric properties similar to human tissue and is often used in phantom tissue simulations [6]. All of the above values were used to calculate a second estimate of the permittivity of rhinoceros dermis *D*_*x*_ as follows:

$$\begin{array}{cc} D_{x} & =(\%water\times \epsilon_{water})+(\%wet\;collagen\times \epsilon_{wet\;collagen})\\ & +(\%dry\;collagen\times \epsilon_{dry\;collagen})+(\%0.1MNaCl\times \epsilon_{0.1MNaCl})\\ & =(60.9\%\times 80)+(33.2\%\times 4.5)+(5.015\%\times 2.3)+(0.885\%\times 78.8)\\ & =51.026725\\ & \approx 51.03 \end{array}$$ (1)

This alternative dermis permittivity approximation will henceforth be referred to as the “Shadwick approximation”. Unlike the values depicted in Table 2, the frequency at which this approximation is appropriate is unknown since it is not specified in the literature. Furthermore, we note that the dermis weighted average approximations in Table 2 of 41.24, 39.79 and 36.41 for the frequencies 403 MHz, 910 MHz and 2.4 GHz respectively are all lower than the Shadwick approximation of 51.03. Since the permittivity values in Table 2 increase with decreasing frequency, the Shadwick approximation seems to correspond to a permittivity measured at a frequency below 403 MHz. In conjunction with the Shadwick dermis approximation *D*_*x*_, the rhinoceros tissue approximations presented in Table 2 provide a range of permittivities that accommodate both the unique mechanical characteristics of rhinoceros skin reported in the literature as well as the extrapolation from animals with similar attributes. Since the dielectric constants of these materials are the ratio between the permittivity (*ε*) and the permittivity of a vacuum (*ε*_0_), these values are referred to as relative permittivities (*ε*/*ε*_0_) and are dimensionless.

All of the tissues have higher conductivity and lower permittivity at higher frequencies. The relative speed with which an electrical signal propagates through tissue is determined by the permittivity of the material. A lower permittivity results in a higher speed of signal propagation, which can be attributed to a higher conductivity and consequent ease with which an electrical current can flow through the material. Thus, since one of the obstacles to the transmission of a signal from an *in vivo* device to an *ex vivo* receiver is the thick skin and fat layer of the rhinoceros, a communication frequency of 2.4 GHz was selected to reduce attenuation effects caused by the resistivity of the material.

### Agar sample measurements

As far as we are aware, no phantom models for rhinoceros tissue have been described in the literature. Therefore, we proceeded with an investigation into various types of gelatinous material commonly used for creating phantom models. We identified agar as the most suitable gelling agent owing to its mechanical properties such as melting and setting temperatures, its non-toxicity and its pliability. Agar recipes were developed for phantom materials that could be used in practical measurements. In total, 75 recipes were developed for the phantom materials of thirteen types of rhinoceros tissue for each of the frequencies of 403 MHz, 910 Mhz, 2.4 GHz and 4.5 GHz. A variety of frequencies were investigated to identify the severity of the effects of non-ideal communication through materials with characteristics similar to rhinoceros tissue. Using 100 ml of each agar mixture, four agar plates were prepared for each recipe in order to allow the calculation of an average permittivity. Thus a total of 300 sample plates were prepared and measured. Usually, such mixtures are placed directly into an autoclave for heating and sterilization. However, due to the large quantities of sugar required by the recipes, the ingredients were dissolved prior to autoclaving using a magnetic stirrer. Air bubbles were removed from the petri dishes while the agar was still warm, since these can influence the dielectric measurements. A broadband measurement system was employed in conjunction with a spectrum analyzer to measure the dielectric properties of the agar samples. Since permittivity is measured relative to air, the permittivity of air was measured after calibrating the probe so that sample permittivity measurements could later be normalised. We found the probe error to consistently be 4.99% (± 0.08%) below the expected permittivity values. The probe error was calculated as the average over two samples of PTFE with the same thickness, each measured a minimum of ten times with probe recalibration before each measurement. Once the configuration of the system was confirmed to be correct, the dielectric properties of the phantom material samples were measured.

### Rhinoceros numerical simulation models

The construction of a physical agar model of a complete rhinoceros was not feasible due to the large size and mass of the animal. Therefore, numerical simulations were used to calculate the effects of electromagnetic propagation. Models of varying detail and complexity were considered, including an anatomical rhinoceros model and a rhinoceros flank model. Each of these models consist of various layers with specific thicknesses and dielectric properties to account for the skin, fat, muscle, blood, organs and skeleton of a rhinoceros. Both the weighted average and the Shadwick permittivity estimates of the rhinoceros dermis were applied to each model for each of the frequencies of 403 MHz, 910 MHz and 2.4 GHz. The computational electromagnetics software FEKO (“**FE**ldberechnung für **K**örper mit beliebiger **O**berfläche”, meaning “field calculations involving bodies of arbitrary shape”) was used to perform the numerical simulations and each layer with its specific dielectric properties was imported as a standard surface mesh. The dimensions used in the anatomical simulation models were based on research published by the International Rhino Foundation [7], Bearcraft and Jamieson [8], Jun Huang [9] and Shadwick et al [3].

#### Anatomical layered model

**Fig 1:**
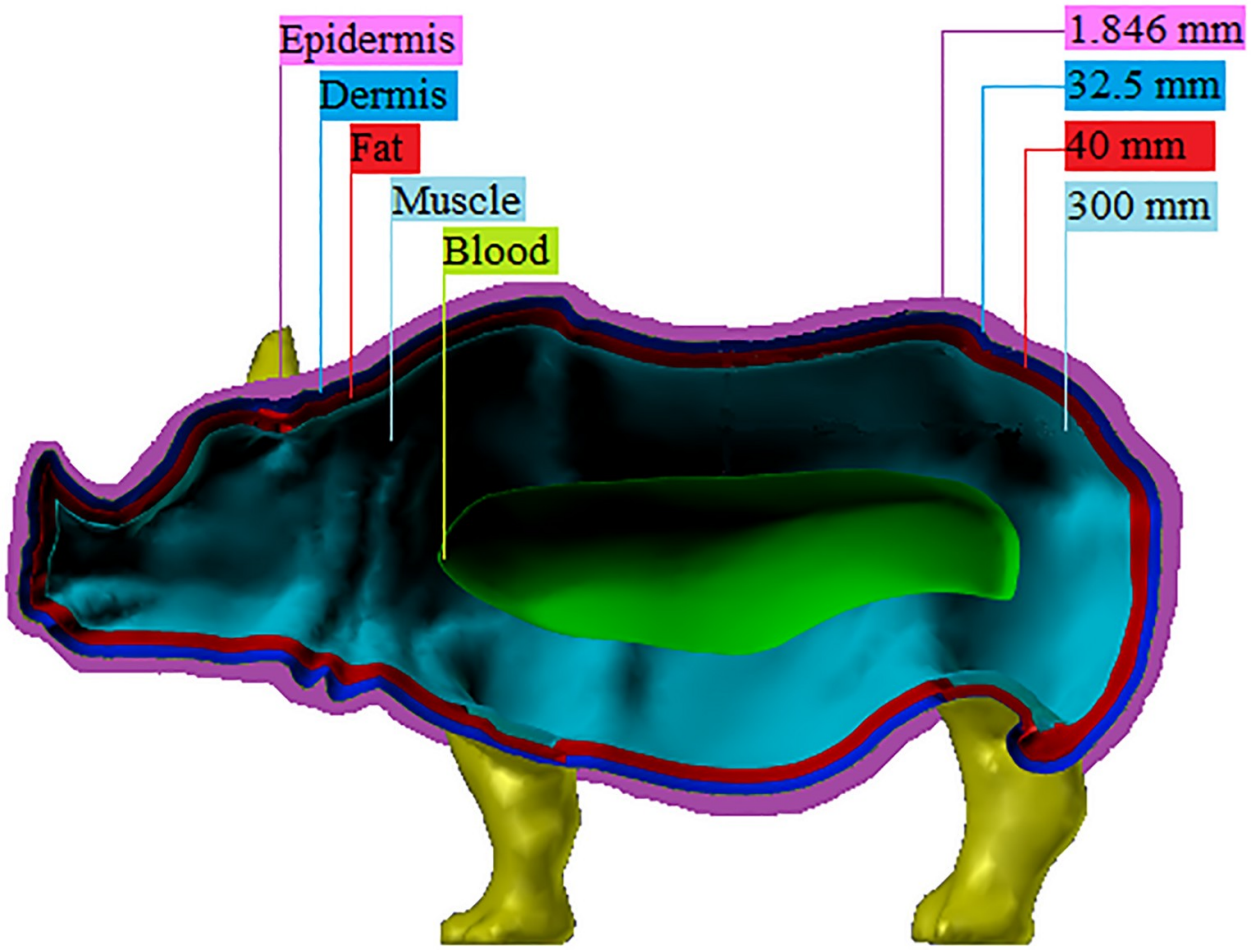
Numerical anatomical layered rhinoceros model. Anatomical layered model of the rhinoceros including a blood layer as a simplified representation of the internal organs. The dielectric properties of each layer are indicated in Table 2.

This model serves as a full-scale rhinoceros approximation. It does not include organs or a skeletal structure, but provides an anatomical approximation of the various layers of rhinoceros skin, such as the epidermis, dermis and fat, and includes muscle and blood layers. The thickness of each layer in the anatomical layered model is indicated in Fig 1. Each layer encapsulates a volume with specific associated permittivity and conductivity and where the innermost volume, referred to as the “blood” layer, provides a simplified representation of the rhinoceros organs.

#### Skeleton and organ models

**Fig 2:**
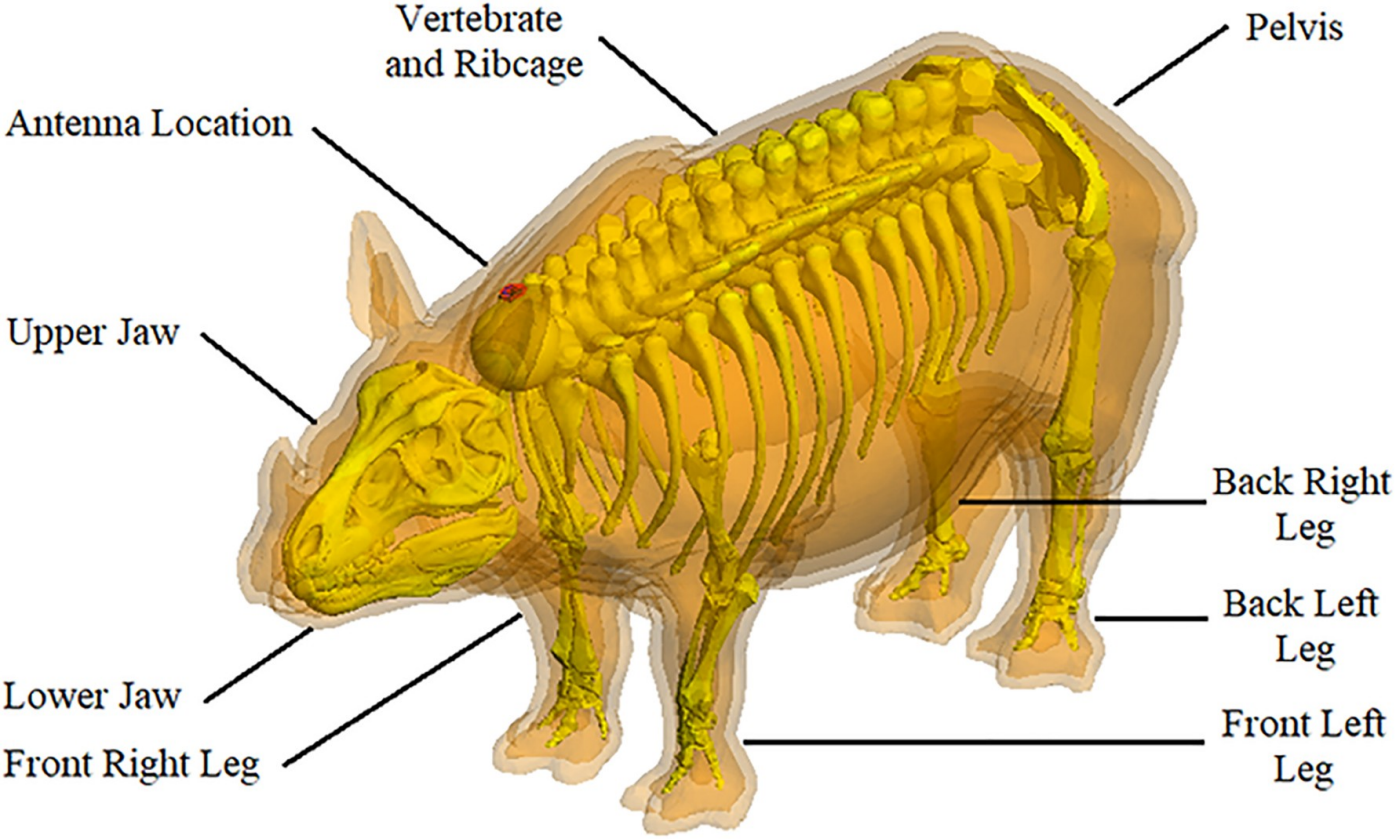
Numerical skeleton and layered anatomical rhinoceros model. Combination of the skeletal and anatomical layered rhinoceros models. The dielectric properties of bone cancellous and bone cortical are indicated in Table 2.

In addition to the layered model described in the previous section, organ and skeletal models were designed for use in the numerical simulations. Since anatomically correct organ and skeletal models are not available for the rhinoceros, these were based on the corresponding geometry of related animals. Specifically, approximations were based on the skeletal structure, organ size and organ location of cattle and horses in accordance with the percentage contributions depicted in Table 1. The dielectric properties of the skeleton and organ models correspond to the bone and blood values in Table 2. The agar recipes matched these permittivity and conductivity values. The skeletal model consists of eight sections as indicated in Fig 2.

Fig 2 illustrates the skeletal model placed within the layered anatomical rhinoceros model and shows how the skeletal model encloses the “blood” layer shown in Fig 1, which is used as an approximation of the organs and does not obstruct the positions of the implantation antennas. Note that these different body parts are not necessarily anatomically accurate for the rhinoceros, but are approximations taken from various animals with similar properties. The size of each skeletal section was scaled to match those of the anatomical layered rhinoceros model.

**Fig 3:**
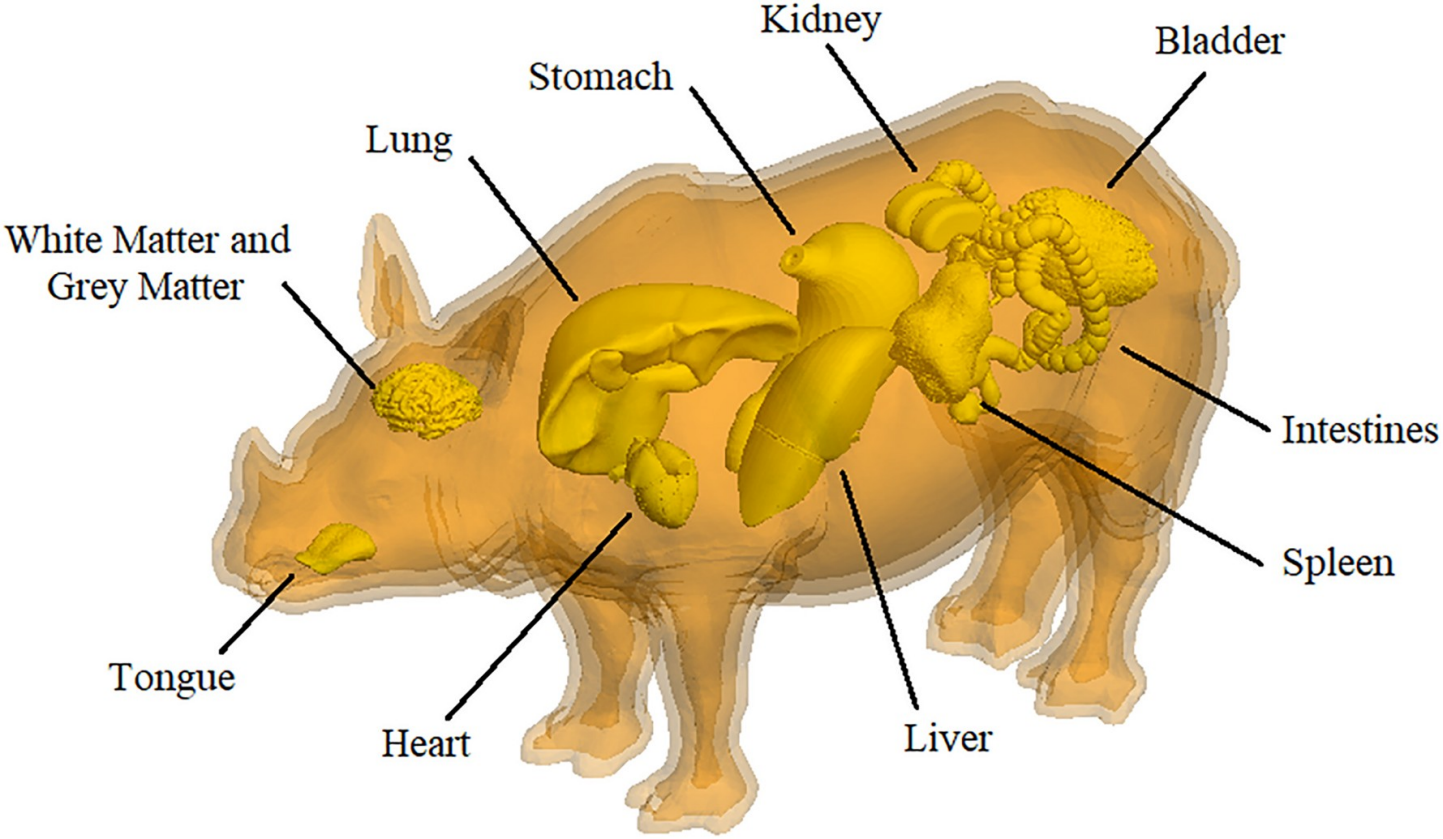
Numerical organ and layered rhinoceros model. Combination of the organ and anatomical layered rhinoceros models. The dielectric properties are as indicated in Table 2.

A geometrical model of the internal organs and tissue layers of the rhinoceros is presented in Fig 3. Again, the implantation positions of the antennas are unimpeded. Some of these geometries were derived from the organs of cattle, but the volumes and locations were primarily based on the internal organs of equine. Due to the previously mentioned scaling factors which are still to be established by means empirical investigation of real rhinoceros organs and tissue, the skeletal model and the organ models illustrated in Figs 2 and 3 are not yet fully compatible in terms of their size and positioning. However, individual organs can be selected and incorporated into the skeletal model as needed. All eleven organ models are independent and have specific dielectric properties as listed in Table 2.

#### Implantation locations

**Fig 4:**
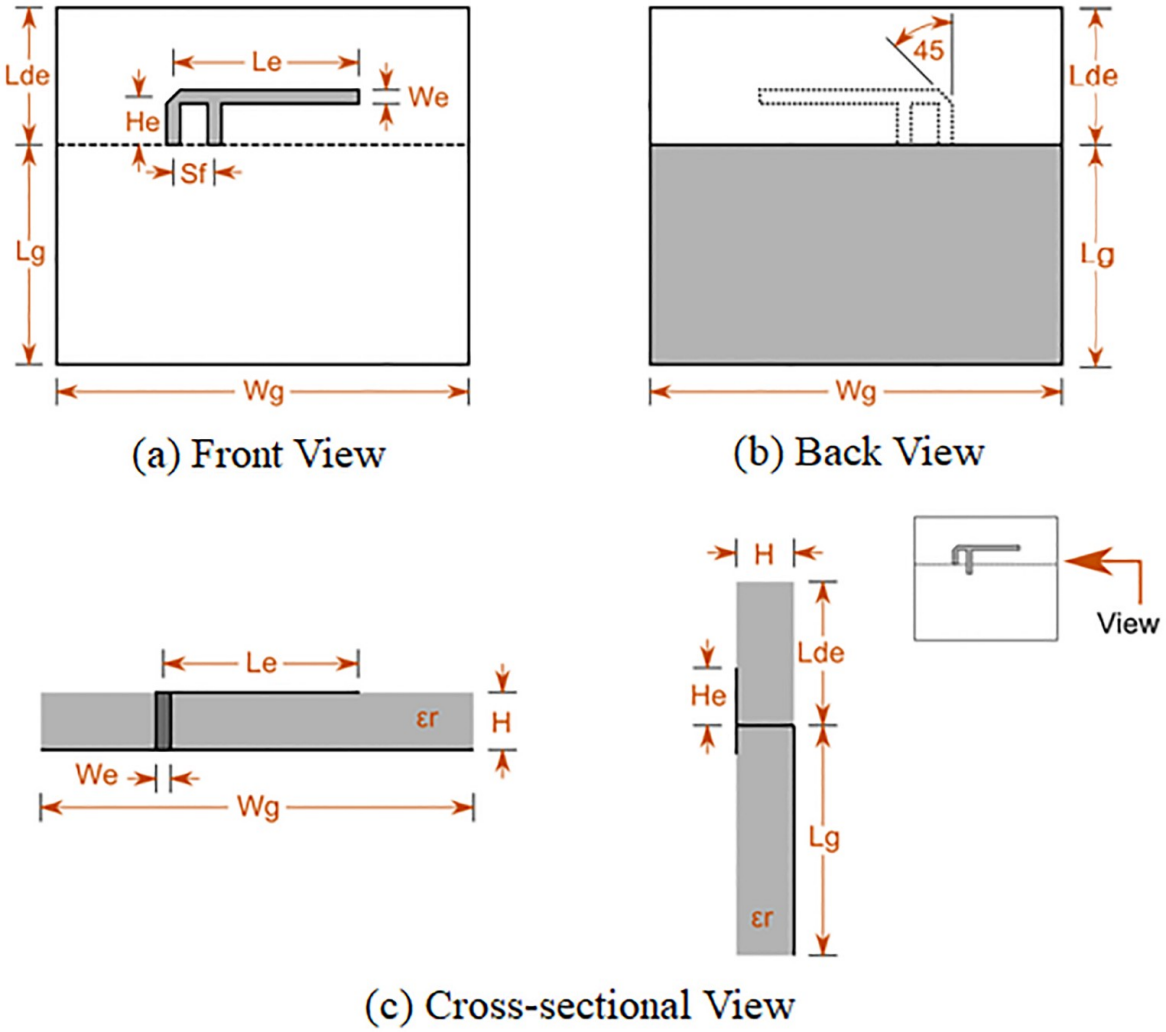
Printed Inverted-F Antenna (PIFA). Typical design and layout of the Printed Inverted-F Antenna (PIFA) which is regularly used in implantation devices.

Potential implantation and antenna design candidates must comply with veterinary specifications. One such specification is a size constraint of 7x5x2 cm for the implantation device and another is a reflection coefficient of less than 0.316. A process of elimination based on these antenna characteristics identified the Microstrip-Fed Planar Elliptical Monopole Antenna (MFPEMA) and the Printed Inverted-F Antenna (PIFA) as suitable candidates for investigation. Antenna size and frequency are inversely proportional and the selection of 2.4 GHz as the frequency of operation reduced the size of the antennas to within the above size constraints. Both these antennas had a return loss of -17.14 dB or less and an omnidirectional propagation pattern, which made them suitable for use in implanted devices. The selected antenna designs were applied to the numerical simulation and agar models to investigate their influence on propagation, power loss and the specific absorption rate (SAR) in the various tissues. A typical PIFA design is illustrated in Fig 4.

**Fig 5:**
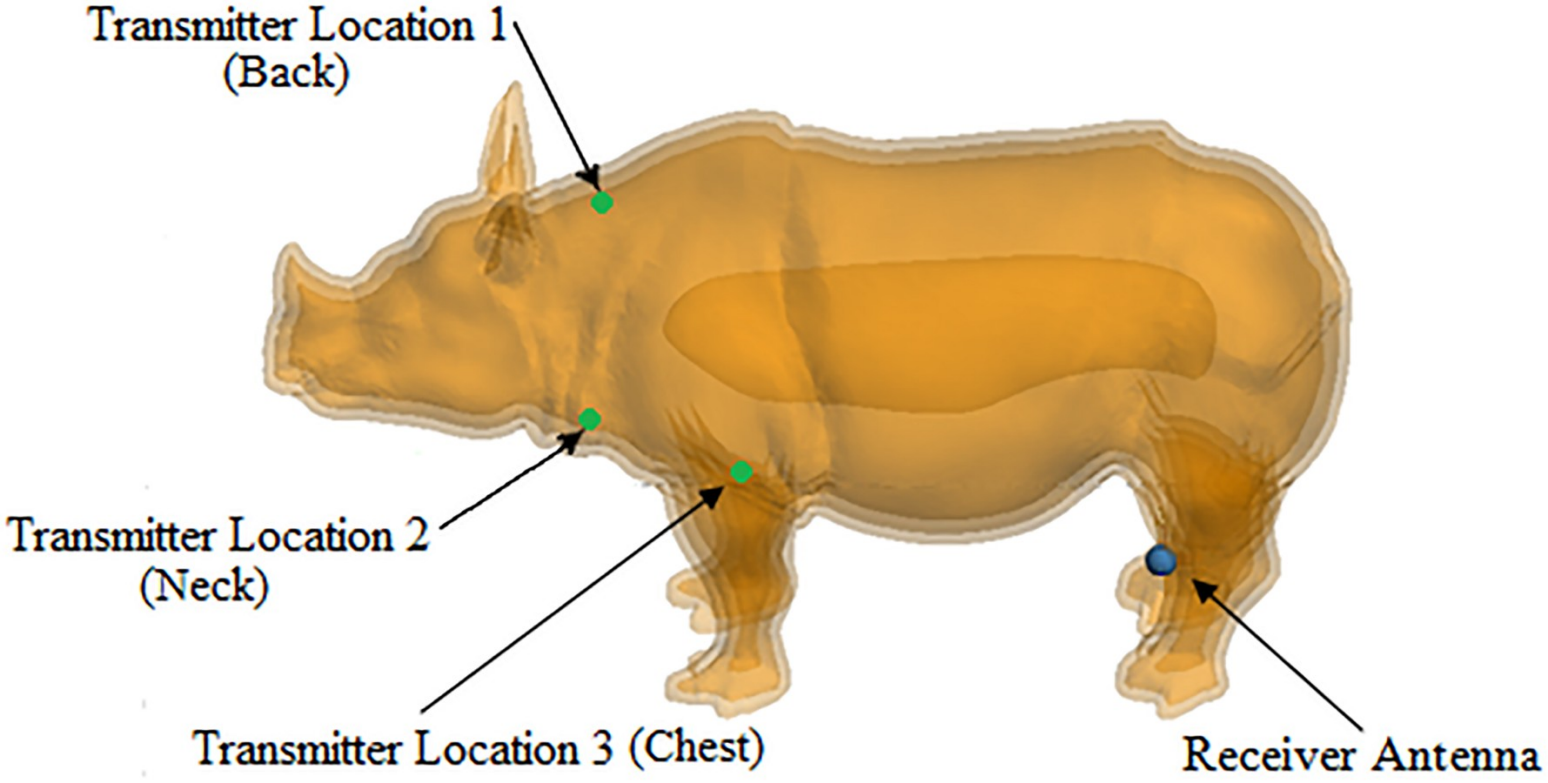
Rhinoceros implant locations. The three considered implantation locations and the location of the *ex vivo* receiver.

The MFPEMA and PIFA were simulated as transmitting and receiving pairs. The transmitting antenna was placed in the fat layer of the anatomical rhinoceros model at the three considered locations, namely in the back, neck and chest regions where each location was tested individually with one transmitter corresponding to one receiver. The receiving antenna was placed outside of the rhinoceros body against the hind left leg. Due to the negligible effect of the epidermis found in initial simulations, this layer was removed to reduce the computational requirements. Fig 5 illustrates the three antenna configurations considered. The direct distance between the *in vivo* back antenna and the *ex vivo* receiving antenna is approximately 2.18 m, which places the receiving antenna within the farfield region according to the Fraunhofer distance. The receiving antenna was also in the farfield region relative to the chest implantation antenna at a direct distance of approximately 1.34 m and 1.95 m relative to the neck implantation antenna. The receiving antenna was positioned in the forward facing direction relative to the rhinoceros for all simulation models. The same directional referencing used in Fig 5 was used for all simulations.

The MFPEMA implanted within the back of the rhinoceros was used to investigate the effect of the skeleton and organs on the power efficiency of the antenna pair at 2.4 GHz. As expected from the theoretical permittivity and conductivity values of bone cancellous and bone cortical tissue, the skeletal model had an insubstantial influence on the propagation characteristics. The SAR peak at the point of implantation was increased slightly by approximately 10 mW/kg and the electric field at the location of the receiving antenna on the hind left leg was reduced by 7 mV/m by including the skeletal model, relative to the anatomical layered model without the skeleton. This reduced the power efficiency of the MFPEMA pair by just 0.08%.

Attempts at numerical simulation using the layered anatomical model including the skeletal model and some of the organ models failed due to insufficient memory on even the largest computing platform available to us (Intel Xeon E7-4850, 2.2 GHz, with 5 TB RAM). However, it was anticipated that these models would also have a minimal effect on the power efficiency of the antenna. This was based on the simulation results of the layered rhinoceros model, which included the blood layer to approximate the organs. Thus, the detailed organ model was not implemented within the layered rhinoceros model in the simulations that follow due to the complexity of their design, which significantly increased the required computational power.

#### Rhinoceros flank phantom model

**Fig 6:**
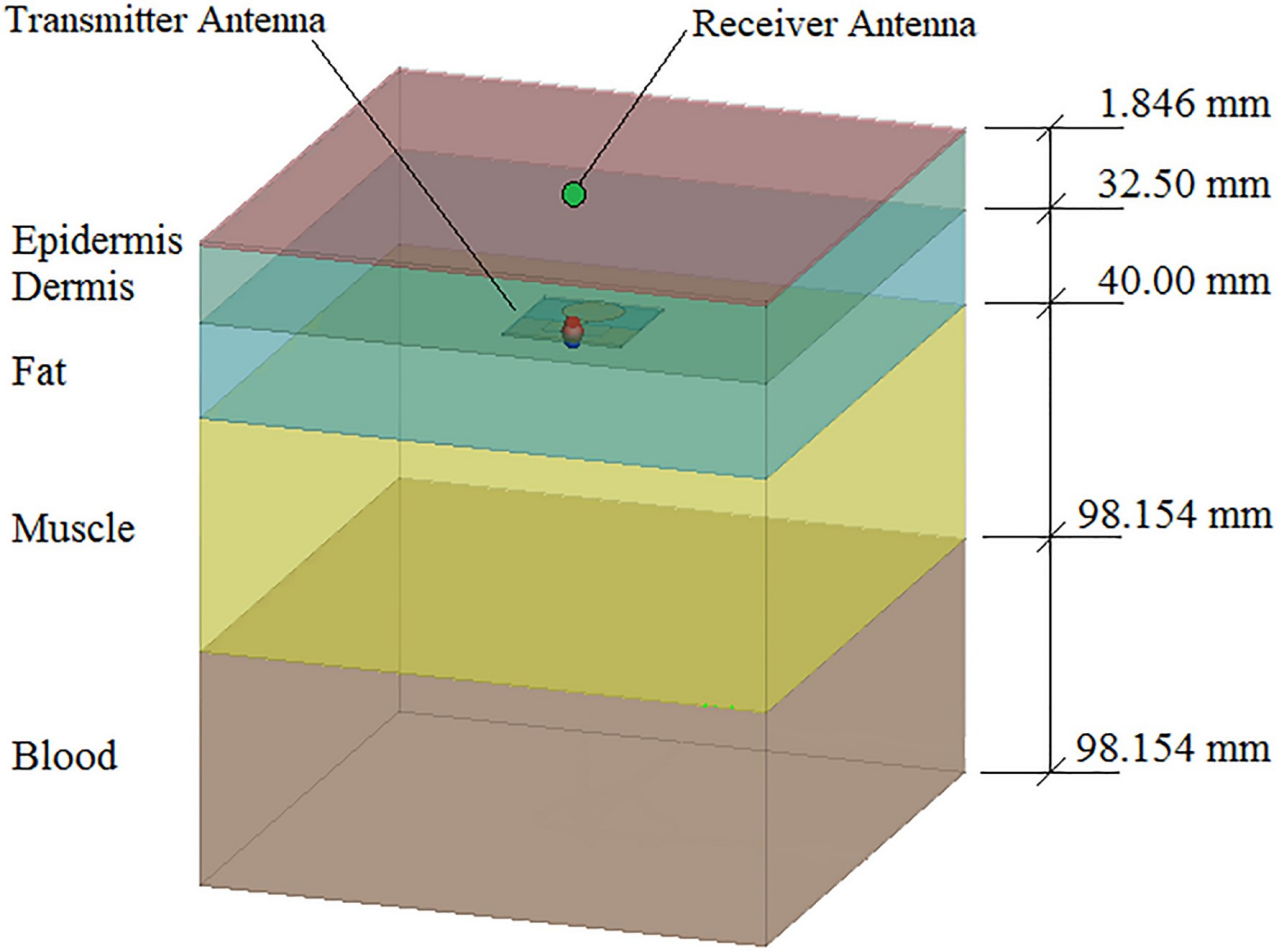
Numerical rhinoceros flank model. Configuration of the rhinoceros flank simulation model with dielectric properties as indicated in Table 2 and a MFPEMA positioned within the fat layer.

This model serves as an inexpensive phantom with manageable size and weight for observing the propagation and attenuation effects of an antenna through various layers of rhinoceros tissue. Specifically, the motivation for constructing the flank model was to validate the accuracy of the simulation in a simplified scenario, so that the simulation results of the anatomical model can be used with confidence. The model can be configured in many ways, for example using the weighted average or Shadwick permittivity approximations. Both approximations were used in simulation and compared with respect to each type of suggested implant antenna at a frequency of 2.4 GHz. This frequency was selected due to its favourable associated antenna size and also because materials generally exhibit an increased conductivity at higher frequencies, which promotes signal propagation and penetration depth. A transmitting antenna was placed facing upward 21.23 cm within the slab model. This locates the antenna in the centre of the fat layer. A receiving antenna was placed facing downward at a height of 27.50 cm, which is just outside and above the slab model. The distance between the antennas was 6.27 cm, which is within the farfield region according to the Fraunhofer distance. The far-field distance is the distance from the transmitting antenna to the beginning of the Fraunhofer region (far field), which was calculated to be 4.16 cm for the 2.4 GHz MFPEMA. Since all of the antennas have a diameter smaller or equal to 5.10 cm, all are situated within the farfield. Fig 6 illustrates the rhinoceros flank phantom model and antenna locations.

## Results

### Rhinoceros flank simulation results

The flank model as shown in Fig 6 was implemented as a numerical simulation. The SAR values indicated that most of the energy (74.4% of the active power) is absorbed by the fat layer in which the antenna is implanted and that the energy dissipates quickly moving away from the antenna position. The peak SAR values of 1.2 W/kg (MFPEMA) and 225 mW/kg (PIFA) are within the stipulated values as regulated by the United States of America, Europe and the IEEE for human tissue. The flank model provided an early indication that transmitting from an *in vivo* antenna in the back, neck or chest to an *ex vivo* antenna located at the hind leg of a rhinoceros, will be challenging. The simulation indicated the power efficiency of the antennas to be 0.18% or less for the Shadwick approximation model and 0.06% or less for the weighted average model, which suggests that wireless charging of rhinoceros implantation devices would be difficult due to the loss of energy through the tissue layers. Since wireless charging was not an explicit aim of this research, this aspect was not further investigated. However, the proposed models could be used in future to explore wireless charging implementations with which to improve the longevity of tracking and monitoring devices.

### Agar sample plate results

Table 3 illustrates the measured permittivity of the 2.4 GHz recipe averaged over four samples and also indicates the error between the measured permittivity and the estimated permittivity given in Table 2. This error will henceforth be referred to as the “Recipe Error”. It can be seen that, for each considered frequency, the recipe error is approximately 5.5% or less with the lowest being close to 3%. Each recipe was executed at least twice and in each case four or more samples were prepared. The average error over the four samples was calculated to yield two permittivity measurements. These permittivity measurements were used to assess the repeatability of the agar preparation process by providing error tolerance levels.

**Table 3:** Measured permittivities of the 2.4 GHz phantom material recipes.

Biological Tissue	Estimated Permittivity	Average Measured Permittivity of Samples	Error [%] (From Avg)	Average Error [%]
**Blood**	54.142	56.639	4.613	4.487
**Grey Matter**	43.852	42.264	3.621	
**White Matter**	32.531	31.176	4.166	
**Kidney**	40.557	39.625	2.299	
**Spleen**	45.829	44.466	2.975	
**Heart**	34.469	32.654	5.264	
**Liver**	39.139	37.161	5.052	
**Lung**	27.567	25.952	5.855	
**Muscle**	53.396	56.377	5.584	
**Skin**	36.405	34.426	5.437	

The estimated (from Table 2) and measured permittivies for the 2.4 GHz rhinoceros phantom agar plates.

Practical measurements were possible for 61 of the 75 proposed recipes with the majority delivering permittivity values below the estimates in Table 2. The recipes that delivered permittivity values higher than the estimates in Table 2 had recipe errors of less than 13.4% (all but one were below 7.0%). Of these recipes, 83.6% had an error margin of 10% or less and even when the most deviant recipes are included, the overall average error (including 403 MHz, 910 MHz and 4.5 GHz) was only 6.2%. The large error associated with the bone and fat recipes was expected, since the literature reports that these materials are difficult to replicate and that they have a large variance with regards to their permittivity [10]. Furthermore, for these tissue types the expected permittivities are generally lower and larger quantities of ingredients are required to achieve the desired dielectric properties. As a result, the solution is closer to its saturation point and when this point is exceeded, the desired permittivity values can not be achieved. This was the case for the 14 recipes for which permittivity measurements were not possible. By selecting the maximum volume and keeping the quantity of the ingredients unchanged, a larger variance is observed compared to the other recipes. The error for the 2.4 GHz recipe group was 4.5%, although the recipes for fat and bone matter delivered liquid state phantom materials. The corresponding error was even smaller for the closest point polynomial regression recipes, with an average recipe error of 4.3%.

**Fig 7:**
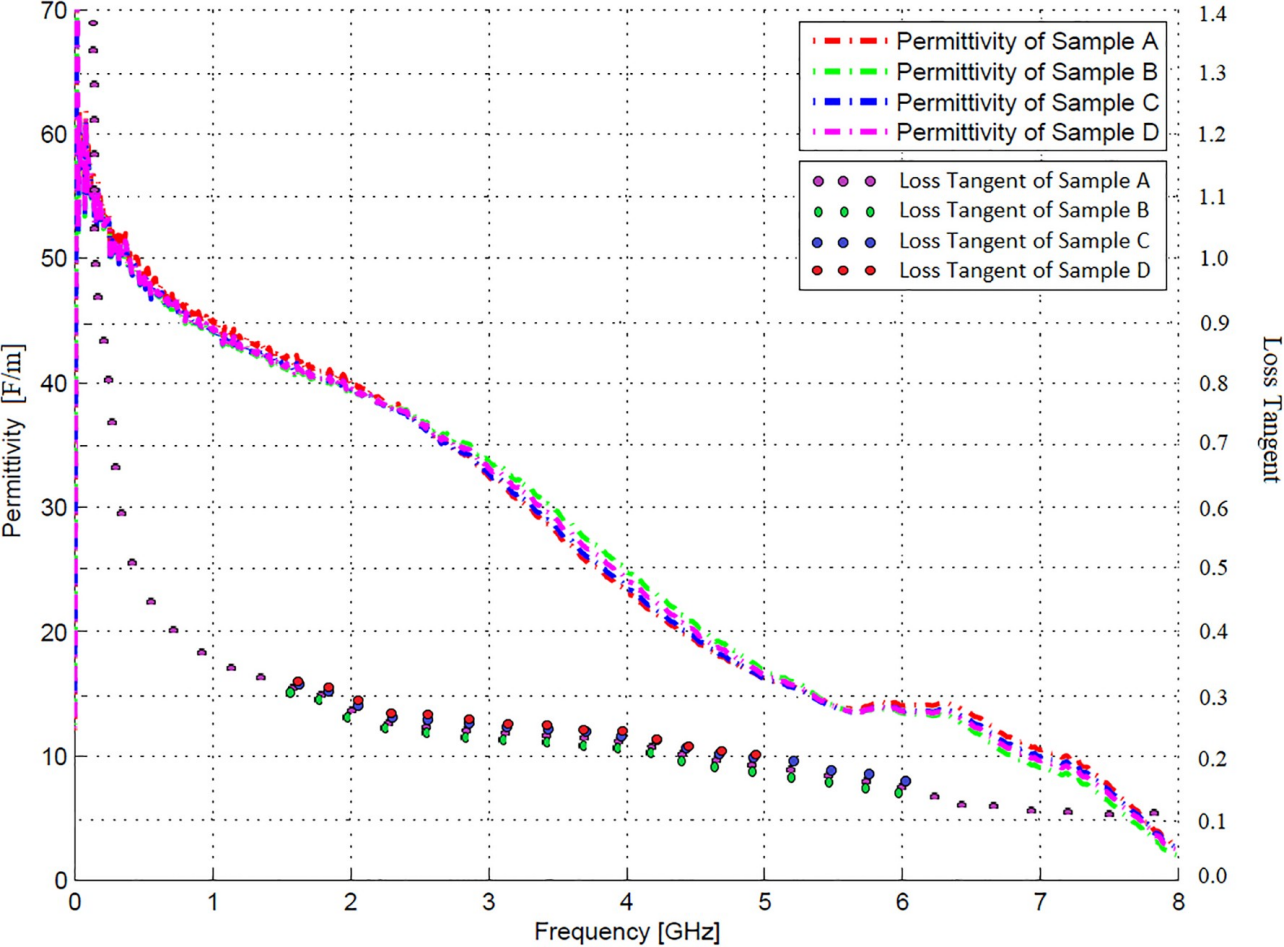
Kidney phantom permittivity measurements. Permittivity and loss tangent measurements of the four 100 ml 2.4 GHz kidney agar phantom recipe samples.

**Fig 8:**
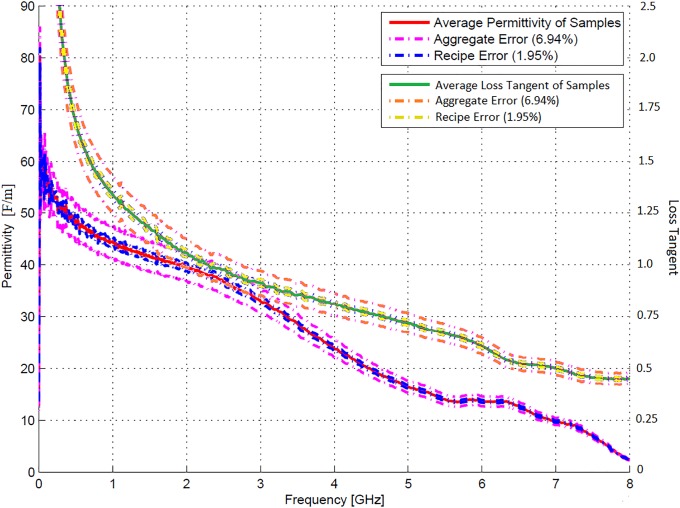
Kidney phantom average permittivity measurements. Average permittivity and loss tangent of the four 100 ml 2.4 GHz kidney agar phantom recipe samples.

The behaviour of phantom tissue permittivity was also investigated in terms of the loss tangent, which is a measure of the rate of electrical energy loss in a system proportional to frequency. The loss tangent was obtained by converting the reflection coefficients to dielectric constants over the frequency range 300 kHz to 8 GHz. Fig 7 illustrates the measured permittivity and loss tangent of the four samples of the 100 ml 2.4 GHz kidney recipe, whereas Fig 8 illustrates the averages of these measurements. The aggregate error, which refers to the variance of the probe, and recipe error margins are also depicted.

Figs 7 and 8 indicate a good agreement between the dielectric properties of the various samples, both in terms of loss tangent and permittivity. Furthermore, the average permittivity is very close to the estimated values in Table 2. A heavy decline was observed in the measured permittivity of some of the samples between 4 and 5 GHz. This is caused by the N-type measurement probe, which is a middle ranged frequency device and could thus only accurately measure the reflection coefficient up to approximately 5 GHz. Since the design frequency of 2.4 GHz is well below this interval, this phenomenon did not influence the measured results.

### Rhinoceros flank phantom model power measurements

The 2.4 GHz rhinoceros agar flank model was constructed and used to investigate the power loss and propagation through the various layers of the phantom materials to establish the similarities and comparability between the agar and simulation models. The MFPEMA and PIFA were used respectively as transmitting/receiving pairs for this purpose.

#### Phantom construction

The model was constructed by pouring the warm liquid phantom material into a mould, layer by layer, to the correct height and allowing it to set. To prevent newly applied liquid agar from melting the previous layer, it was first cooled to approximately 55°C. This is warm enough to keep the phantom material in a liquid state, but cool enough to avoid liquifying the phantom material once it has set. This method also avoided the formation of air gaps between the layers. It however does not allow for the individual testing of the layers and the antenna can only be inserted by means of an incision.

#### Antenna placement

**Fig 9:**
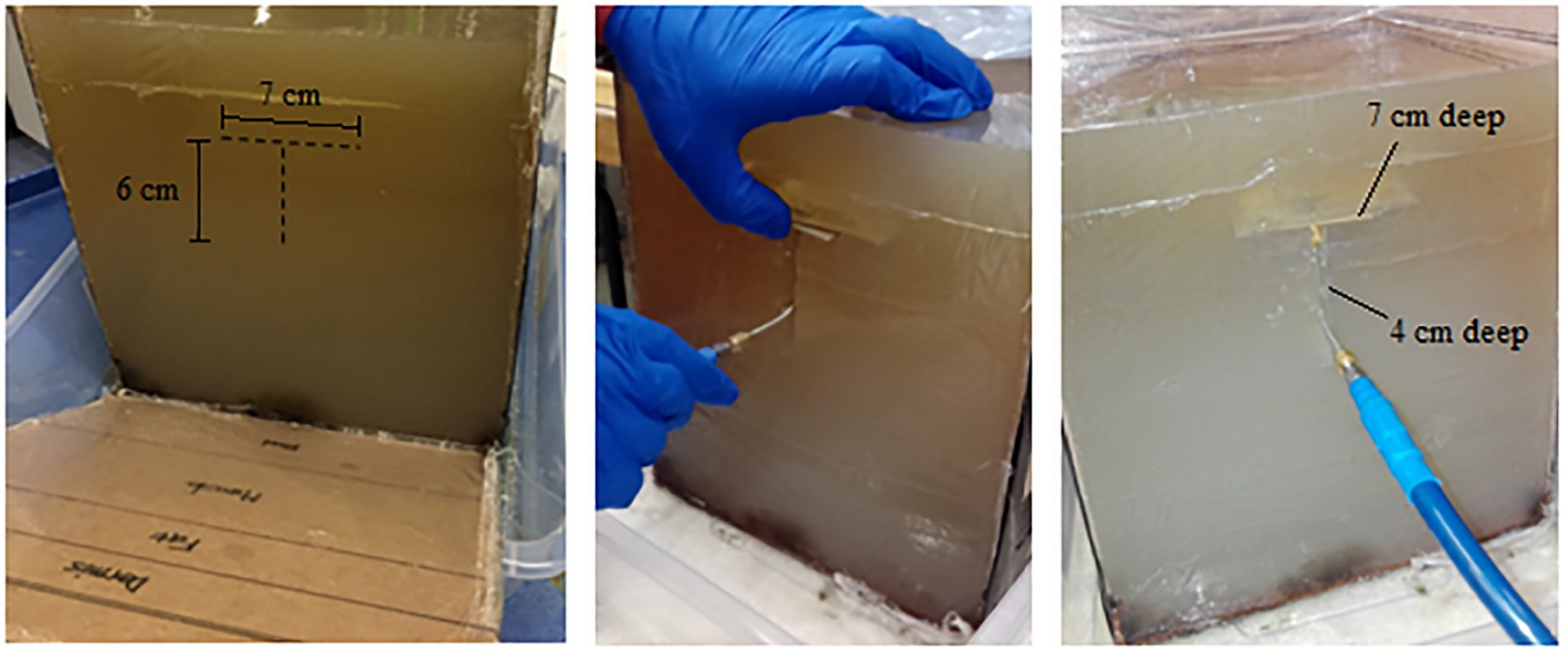
Practical rhinoceros flank phantom incision. T-incision on the side of the 2.4 GHz rhinoceros phantom flank model used to insert the antenna for practical measurement.

The distance between the implanted antenna (transmitter) and the *ex vivo* antenna (receiver) was 5.43 cm. These distances were based on the expected thickness of rhinoceros tissue. Foam was used to simulate the air gap between the implanted antenna and the casing of the implantation device. The cable loss at 2.4 GHz was measured to be -1.04 dBm using a spectrum analyzer. The power loss through an air gap of 5.43 cm was measured to be -15.69 dBm for the MFPEMA pair and -22.50 dBm for the PIFA pair. The same measurements were conducted using the agar rhinoceros flank model. This required the transmitting antenna to be placed within the agar phantom, which was achieved by means of an ‘T’ incision as illustrated in Fig 9. The upper horizontal incision of the T-shape was 5.43 cm from the top of the flank model, which places it exactly in the middle of the fat layer. The horizontal cut was 7 cm long and 7 cm deep to accommodate all considered antennas. The vertical cut, which was situated in the centre of the flank model, was 6 cm long and 4 cm deep to provide an entry point for the connector cable.

**Fig 10:**
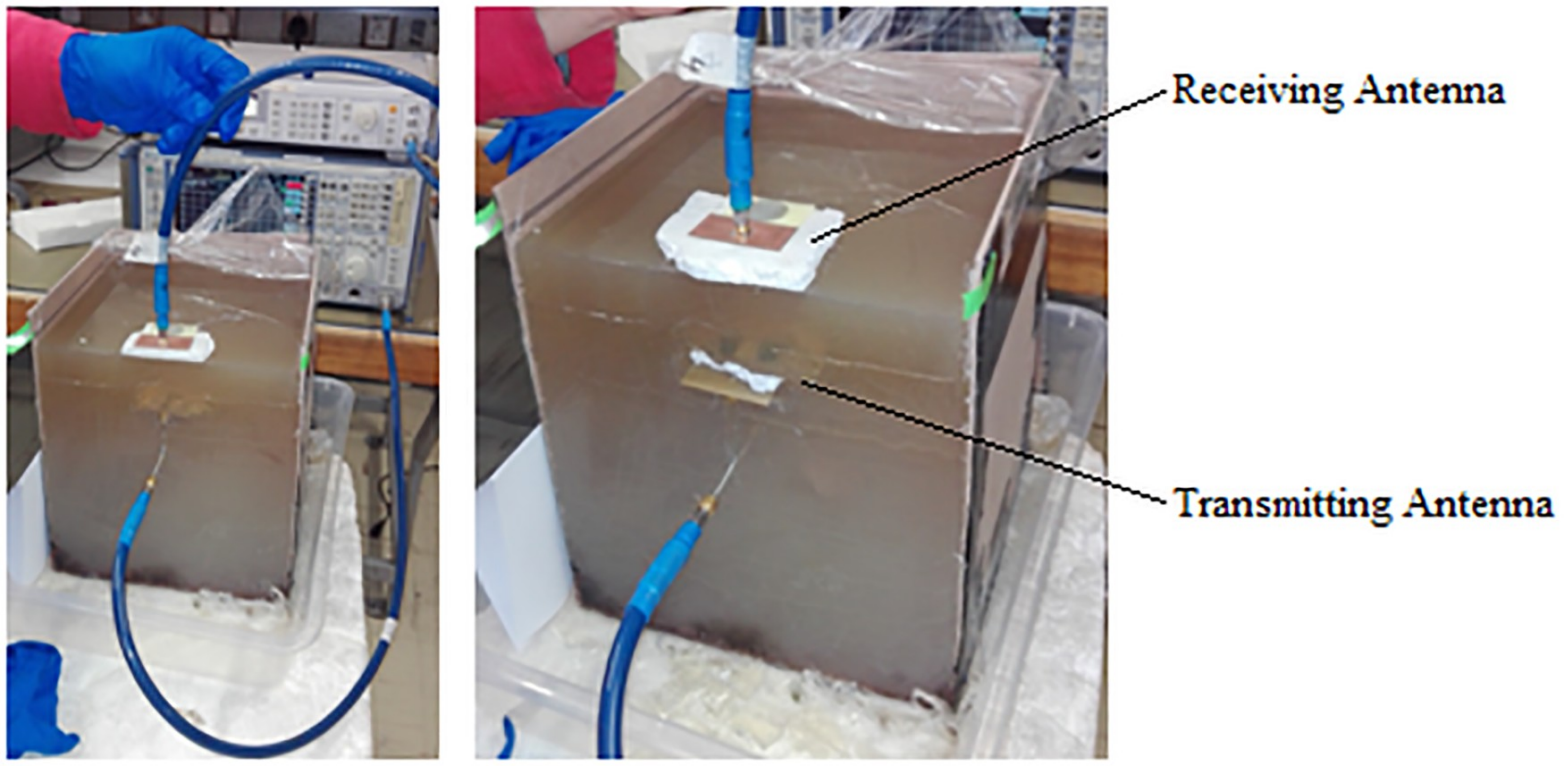
Practical rhinoceros flank phantom power measurement. The configuration of the power loss measurement of the 2.4 GHz rhinoceros flank model.

Losses in the cable feeding the implanted antenna are expected to increase with length and thus the implanted antenna was not placed deeper within the phantom model. A piece of foam 10 mm thick was attached to the transmitting and receiving antennas to simulate the air gap that would exist between the implanted antenna and its casing. Measurements were taken with and without the foam air gap in order to evaluate the losses caused by direct contact with the agar. Further investigation was conducted by placing the receiving antenna at an 20 mm offset (in the x- and y-directions) relative to the implanted MFPEMA, in order to establish whether or not such nonalignment would have a significant mitigating effect on propagation. This reflects the movement of the *in vivo* antenna relative to the *ex vivo* receiver, as it could migrate before settling within the fat layer. Fig 10 illustrates the practical configuration of the power loss measurement of the 2.4 GHz rhinoceros flank model.

### Rhinoceros flank power measurement results

Table 4 presents the power loss measurements for the various antennas (cable loss excluded). An improvement in power efficiency with the introduction of the air gap between the antenna and the agar phantom is clearly discernable. The nonalignment of the antennas also seems to cause a slight reduction in the received power, based on the MFPEMA measurement. The experiment was repeated for the MFPEMA and PIFA pairs respectively, using the same input power indicated by the corresponding numerical simulations. The loss of the cable connecting the transmitting antenna to the network analyzer was measured as -1.15 dBm and the loss of the cable connecting the receiving antenna to the network anaylzer was measured as -0.8 dBm at 2.4 GHz, with an output of 11.60 dBm. The MFPEMA requires an output of 11.61 dBm. Thus, taking the cable loss into account, the output was adjusted to 12.75 dBm. Similarly, the output was adjusted to 12.12 dBm for the PIFA measurements in order to match the required 10.97 dBm output observed in the simulations.

**Table 4:** Received power through the 2.4 GHz rhinoceros flank phantom.

**Input power = 0 dBm**			
**Antenna**	**Direct Contact**	**10 mm Foam**	**20 mm Nonalignment**
**MFPEMA**	-74.92 dBm	-53.96 dBm	-58.94 dBm
**PIFA**	-61.95 dBm	-58.91 dBm	-
**Input power = +12 dBm**			
**Antenna**	**Direct Contact**	**10 mm Foam**	**Air Gap (54.346 mm)**
**MFPEMA**	-58.60 dBm	-42.03 dBm	-3.50 dBm
**PIFA**	-52.70 dBm	-36.23 dBm	-8.70 dBm

The measured power through the 2.4 GHz rhinoceros flank phantom as received by the *ex vivo* antenna.

The second part of Table 4 depicts the power measurements of the MFPEMA and PIFA pairs through the flank model using parameters similar to those in the simulations. Once again, the results indicate an improvement in power efficiency with the introduction of the 10 mm air gap between the antenna and the agar phantom. The measured weight of the rhinoceros flank model was approximately 20 kg, which is slightly more than the theoretical estimate of 17.16 kg. Comparing the measurements in Table 4 with the numerical simulation results, it is clear that the higher density of the heavier physical agar compared to the theoretical estimations had a greater attenuating effect on the propagation pattern of the antenna. As for the simulations, the PIFA has a slightly higher power efficiency than the MFPEMA.

The simulations exhibit ideal circumstances and do not incorporate the effects of non-ideal contact between the antenna and the agar, or of cable loss, impurities within the agar and the effect of air particles. The air particles are represented by a lossless medium (free space). Thus, additional mitigation effects were added to the flank model. This included applying the measured recipe errors of the dielectric properties of each layer to the flank model and a small attenuation factor to the air medium used in the simulations. This reduced the power received by the *ex vivo* antenna and increased the comparability of the power efficiency of the theoretical and practical models to 67.4%.

Factors such as the density and temperature of the medium are difficult to incorporate accurately into a simulation model, because their effect on signal propagation is difficult to quantify. However, we have found their inclusion to be critical and that without them, good agreement between practice and simulation can not be achieved. The correspondence between simulation and practice could be further enhanced by identifying and applying more of these influences. Nevertheless, the good correspondence already achieved allows us to conclude that our simulations are a useful representation of reality and can be used as a basis for design decisions.

### Rhinoceros anatomical phantom simulation results

The anatomical rhinoceros model described earlier, including the blood layer which is an approximation of the internal organs, was used to investigate the feasibility of communication between an implanted transmitter and an *ex vivo* receiver attached to the hind leg. Two antenna pairs (MFPEMA and PIFA respectively) and three implant locations were considered. In each case, both the weighted average and the Shadwick dermis dielectric approximations were applied. Six configurations were used to investigate the propagation characteristics of the antennas: the Shadwick and weighted average approximations for each of the back, chest and neck configurations. In all cases the organs and skeleton were excluded from the anatomical rhinoceros model. For each configuration, the gain, SAR, electric field and received power were considered.

#### MFPEMA transmitting and receiving pair

It is clear from both dermis approximations that the propagation towards the rear of the rhinoceros is severely attenuated and that most of the energy escapes in the forward facing direction of the rhinoceros. The maximum gain is approximately -23 dBi for the Shadwick approximation and -27 dBi for the weighted average approximation when the implant is located in the back, and -26 dBi for the Shadwick approximation and -33 dBi for the weighted average approximation when the implant is located in the neck. These results agree with the earlier findings which suggested that the weighted average model has a greater attenuating effect on the antenna radiation.

**Fig 11:**
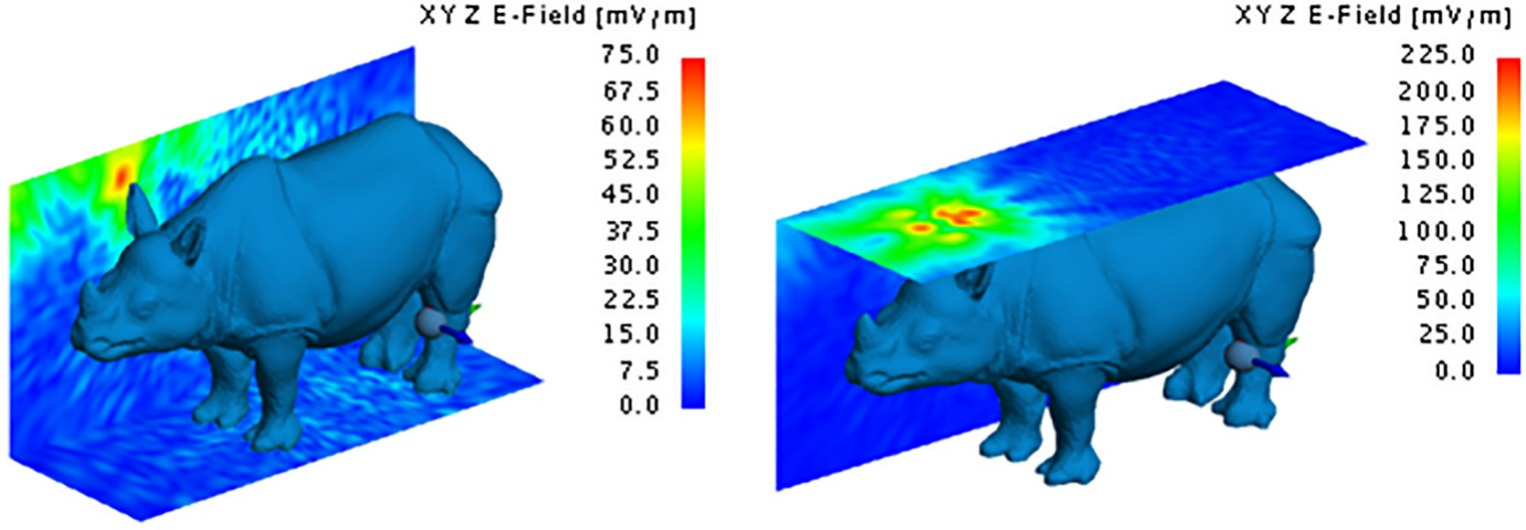
Electric field of the MFPEMA implanted in the back with the Shadwick dermis approximation. The electric field of an MFPEMA transmitting and receiving pair, when the implantation is located in the back and the Shadwick dermis approximation is applied.

**Fig 12:**
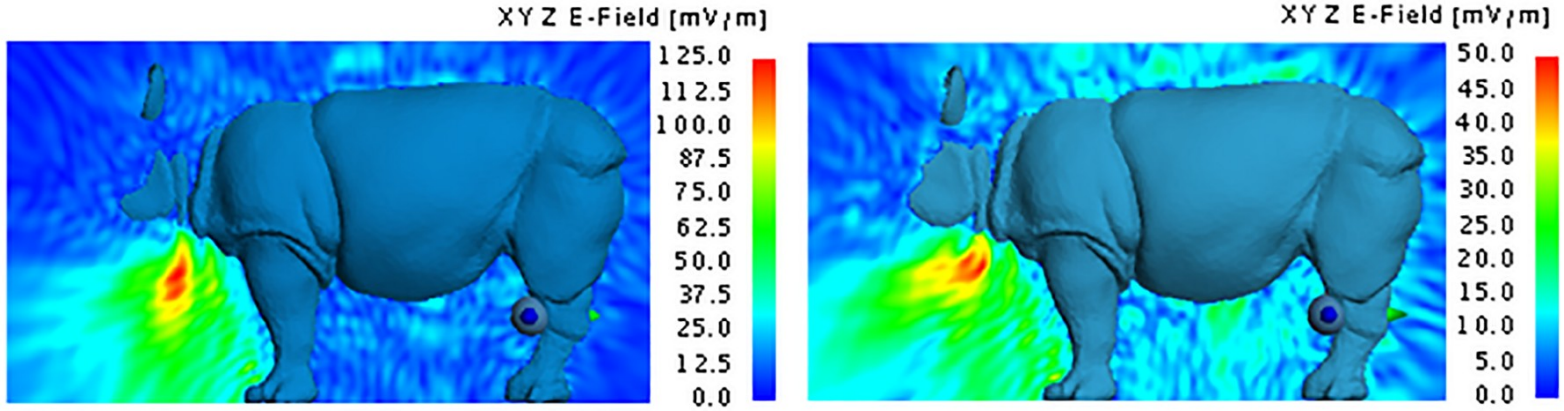
Electric field of the MFPEMA implanted in the neck. The electric field of the MFPEMA transmitting and receiving pair, when the implantation is located in the neck and the Shadwick [left] and weighted average [right] dermis approximations are applied.

**Fig 13:**
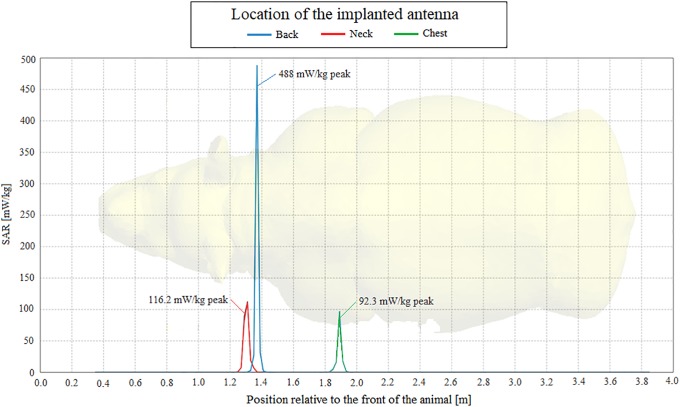
Specific absorption rate of the MFPEMA using the Shadwick dermis approximation.

**Fig 14:**
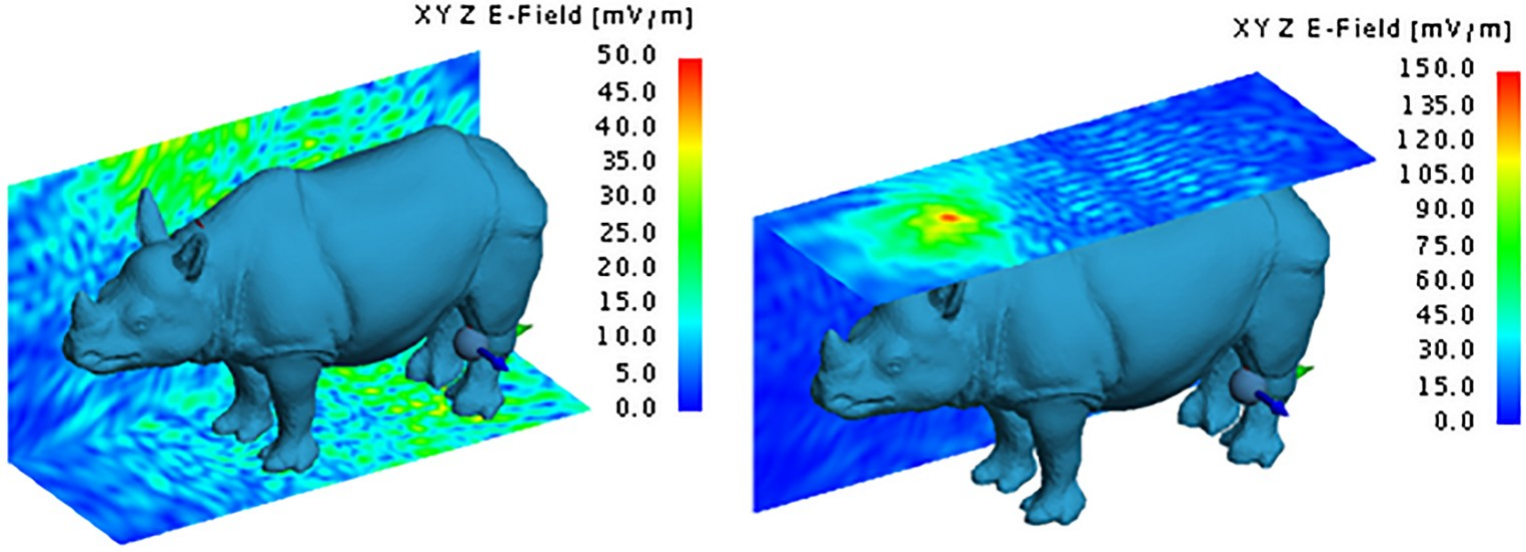
Electric field of the PIFA implanted in the back. The electric field of a PIFA transmitting and receiving pair, when the implantation is located in the back and the Shadwick dermis approximation is applied.

**Fig 15:**
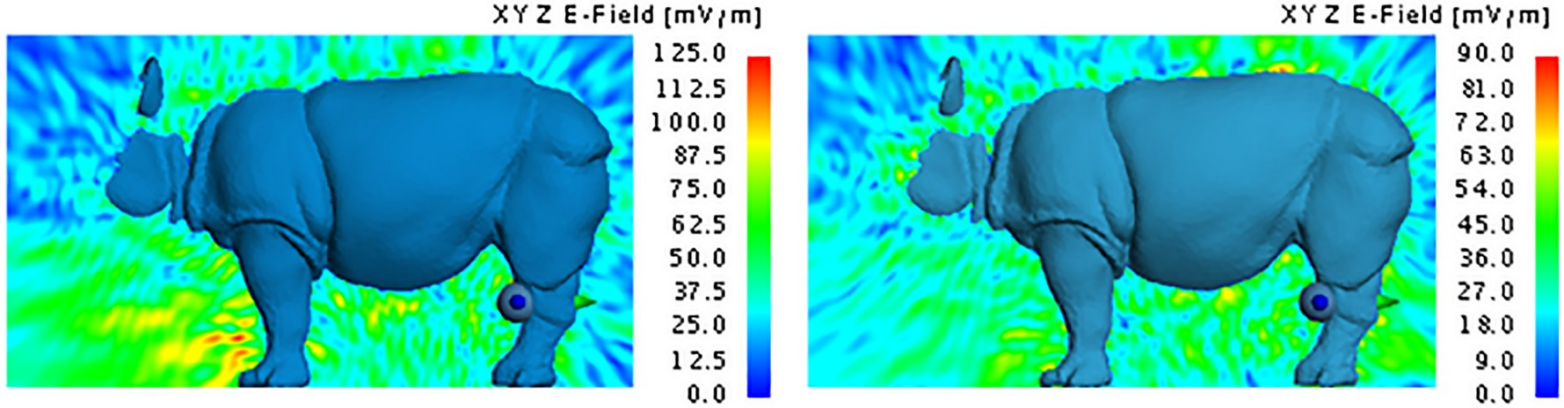
Electric field of the PIFA implanted in the neck. The electric field of a PIFA transmitting and receiving pair, when the implantation is located in the neck and the Shadwick [left] and weighted average [right] dermis approximations are applied.

The dispersion of the electrical field around the rhinoceros gives a visual representation of the way in which the transmitted energy dissipates from the implanted location. Figs 11 to 15 respectively illustrate the yz and xy behaviour of the electric field when using the Shadwick and weighted average approximations for the back and neck antenna locations. Once again, it is clear that very little energy penetrates the thick hide of the rhinoceros. The maximum electric field values were 210 mV/m and 108 mV/m for the Shadwick and weighted average approximations respectively at the location of the back implant, and 87.5 mV/m and 32 mV/m for the Shadwick and weighted average approximations respectively at the location of the neck implant.

Although a large difference was observed between the two dermis approximations at points close to the implantation site, both models indicated a similar electric field at the location of the receiving antenna, as illustrated by Fig 12. For an implantation in the back, the approximate electric field at the receiver on the hind leg was 30 mV/m for the Shadwick approximation and 27 mV/m for the weighted average approximation. These values decreased to 25 mV/m for the Shadwick approximation and 12 mV/m for the weighted average approximation for the neck implantation. The indicated yz-surface is located 27.2 cm from the x-position of the implantation antenna and cuts through the position of the receiving antenna. These illustrations support the notion that, although the implanted antennas are capable of penetrating the thick hide of the rhinoceros, they are not effective at transmitting a signal from an *in vivo* position at the back of the neck to an *ex vivo* location at the hind leg. This is confirmed by the received power at the hind leg, which is a mere 149.86 pW for the Shadwick approximation and 214.44 pW for the weighted average approximation, as calculated by simulation. A similar received power was attained for the neck configurations: 194 pW for the Shadwick approximation and 2.47 pW for the weighted average approximation.

Fig 13 shows the SAR lengthwise through the rhinoceros model. It is evident that most of the energy is absorbed within a few centimetres of the point of implantation and that the positions of the SAR peaks coincide with the positions of the implanted antennas. The SAR values of 488 mW/kg and 495 mW/kg respectively for the Shadwick and weighted average approximations and the back implantation were the highest among all considered model configurations. These values are nevertheless within the acceptable range of SAR exposure for human tissue as regulated by the IEEE and ICNIRP. Corresponding exposure limits are not currently available for rhinoceros. Based on the total active power of approximately 14.6 mW, the simulation determined the efficiency of the MFPEMA as 0.13% or less for the Shadwick approximation and 0.05% or less for the weighted average approximation. It was apparent that most of the energy was absorbed by the fat layer (>10 mW), followed by the dermis and the muscle layers. The power loss experienced in these layers was corroborated by the specific absorption rates, which indicate similar trends with the highest power loss in mediums with the highest absorption rates due to their lower permittivity and conductivity. The SAR of the models using the weighted average dermis approximation was very similar to the SAR when using the Shadwick dermis approximation illustrated in Fig 13. The SAR values for the weighted average dermis approximation with the implant located in the back, neck and chest were 495 mW/kg, 114.1 mW/kg and 89.5 mW/kg respectively.

#### PIFA transmitting and receiving pair

Numerical simulations were performed with the PIFA placed in the back of the anatomical layered rhinoceros model. Once again, propagation towards the rear of the rhinoceros experiences severe attenuation and most of the energy escapes in the forward facing direction. The maximum gains are approximately -26 dBi, -24 dBi and -25 dBi with the implant located in the back, chest and neck respectively for the Shadwick appoximation and -28 dBi, -26 dBi and -27 dBi for the weighted average appoximation. The input power to the transmitting antenna was approximately 12.6 mW in all cases. These results agree with the earlier findings suggesing that the weighted average dermis approximation has a greater attenuating effect on the antenna radiation.

The dispersion of the electrical field at the same locations considered for the MFPEMA was similar, as illustrated in Fig 14. Once again, it is clear that very little energy penetrates the thick hide of the rhinoceros. The maximum electric field strength at the points of implantation were 235 mV/m, 140 mV/m and 85 mV/m when the implant was located in the back, chest and neck respectively and when using the Shadwick configuration. The corresponding values were 135 mV/m, 75 mV/m and 80 mV/m respectively when using the weighted average appoximation. The electric field close to the location of the implant tends to be lower for the weighted average tissue approximation than for the Shadwick tissue approximation when the implant is in the back and chest locations. However, similar electric fields were observed at the location of the receiving antenna, as illustrated by Fig 15. Here the approximate field strengths were 55 mV/m, 55 mV/m and 70 mV/m when the implant is located in the back, chest and neck respectively for the Shadwick approximation and 32 mV/m, 45 mV/m and 50 mV/m respectively for the weighted average approximation. The yz-surface is located 27.2 cm from the x-position of the implantation antenna and cuts through the position of the receiving antenna.

**Fig 16:**
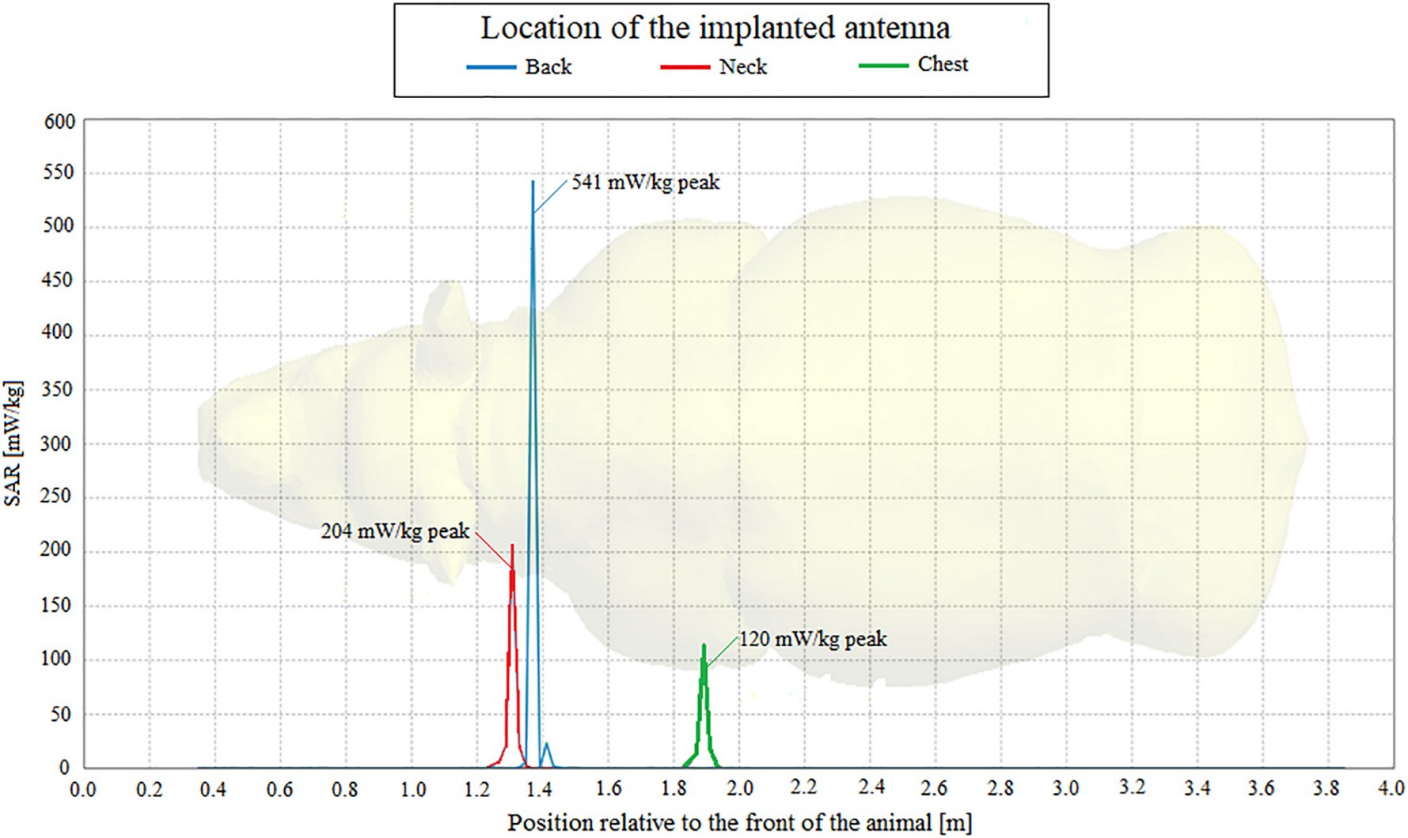
Specific absorption rate of the PIFA using the Shadwick dermis approximation. SAR of the PIFA propagating lengthwise through the rhinoceros for back, chest and neck implantation locationswhen using the Shadwick dermis approximation.

These analyses support the notion that, although the implanted antennas are capable of penetrating the thick hide of the rhinoceros, transmission of a signal from an *in vivo* position to an *ex vivo* location at the hind leg is challenging but possible. The received powers of 512.8 pW, 1.6 nW and 850.7 pW with the implant located in the back, chest and neck locations respectively for the Shadwick appoximation and 564.2 pW, 1.8 nW and 1.7 nW for the weighted average appoximation allow us to conclude that the chest is the best of the three considered implantation locations if the objective is communication with an *ex vivo* device attached to the hind leg. Fig 16 illustrates the SAR lengthwise through the rhinoceros model using Shadwick dermis approximation. It can be seen that most of the energy is absorbed within a few centimetres of the point of implantation. Again, the SAR when using the weighted average dermis approximation was very similar to the SAR when using the Shadwick dermis approximation illustrated in Fig 16. The SAR values when using the weighted average dermis approximation with the implant located in the back, neck and chest were 553 mW/kg, 205.5 mW/kg and 116 mW/kg respectively.

The maximum SAR values of 541 mW/kg and 553 mW/kg, shown in Fig 16, were attained using the Shadwick and weighted average appoximations respectively and when the implant was located in the back. These and the specific absorption rates per tissue type presented in Table 5 are within acceptable human radiation exposure limits as regulated by the IEEE and ICNIRP. Corresponding exposure limits are not currently available for rhinoceros. These power losses per tissue were determined by simulatiuon with the PIFA located in the back, chest and neck respectively, and indicate that the peripheral tissue layers of the rhinoceros are most prohibitive to signal propagation when the antenna is positioned within the fat layer.

**Table 5:** Simulated specific absorption rate and power loss of the individual phantom layers (PIFA).

**Back Implantation**				
**Biological Tissue**	**Shadwick Dermis Approximation**		**Weighted Average Dermis Approximation**	
	**Specific Absorption Rate [W/kg]**	**Power Loss [W]**	**Specific Absorption Rate [W/kg]**	**Power Loss [W]**
**Dermis**	2.4321E-06	1.1745E-03	2.8235E-06	1.3635E-03
**Fat**	19.4576E-06	9.7067E-03	19.7723E-06	9.8637E-03
**Muscle**	0.4272E-06	0.7175E-03	0.4606E-06	0.7736E-03
**Blood**	4.5788E-17	8.3272E-15	9.5966E-18	1.7453E-15
**Average SAR for entire domain/Sum of all losses**	4.0794E-06	12.5050E-03	4.2208E-06	12.9450E-03
**Chest Implantation**				
**Biological Tissue**	**Shadwick Dermis Approximation**		**Weighted Average Dermis Approximation**	
	**Specific Absorption Rate [W/kg]**	**Power Loss [W]**	**Specific Absorption Rate [W/kg]**	**Power Loss [W]**
**Dermis**	2.1456E-06	1.0362E-03	2.4765E-06	1.1960E-03
**Fat**	19.7517E-06	9.8534E-03	19.5345E-06	9.7450E-03
**Muscle**	0.5092E-06	0.8552E-03	0.4961E-06	0.8332E-03
**Blood**	9.7760E-16	1.7779E-13	1.4748E-17	2.6821E-15
**Average SAR for entire domain/Sum of all losses**	4.1307E-06	12.6360E-03	4.1411E-06	12.6750E-03
**Neck Implantation**				
**Biological Tissue**	**Shadwick Dermis Approximation**		**Weighted Average Dermis Approximation**	
	**Specific Absorption Rate [W/kg]**	**Power Loss [W]**	**Specific Absorption Rate [W/kg]**	**Power Loss [W]**
**Dermis**	2.5446E-06	1.2288E-03	2.7335E-06	1.3201E-03
**Fat**	19.1536E-06	9.5550E-03	19.2035E-06	9.5799E-03
**Muscle**	0.5675E-06	0.9532E-03	0.5729E-06	0.9622E-03
**Blood**	5.9628E-15	1.0844E-12	1.6932E-15	3.0793E-13
**Average SAR for entire domain/Sum of all losses**	4.1281E-06	12.5580E-03	4.1721E-06	12.6840E-03

Numerical results of the SAR and power loss of the PIFA.

Based on the total active power of approximately 12.6 mW, the simulations indicate that the efficiency of the PIFA is 0.23% or less when using the Shadwick appoximation and 0.15% or less when using the weighted average appoximation. Again, most power is absorbed by the fat layer (> 9.5 mW), followed by the dermis and the muscle layers. These findings suggest that the PIFA has a slightly higher power efficiency than the MFPEMA.

## Discussion

We have presented phantom and numerical simulation models which mimic the dielectric properties of a rhinoceros and which can be used to investigate the design of *in vivo* and *ex vivo* devices as are, for example, required for animal tracking and monitoring. Two numerical models were designed to investigate the propagation characteristics of implanted antennas: a rhinoceros flank model and an anatomical rhinoceros model. The proposed flank model, which represents part of the loin of the rhinoceros, includes all the layers of the anatomical rhinoceros model and served to verify the accuracy of the proposed numerical model by allowing comparison with practical measurements. The comparability of the power efficiency of the theoretical and practical models was good (67.38%) and indicates that the model is a sufficient representation of rhinoceros tissue for practical use. The flank model simulations and practical measurements also confirmed that penetration of the thick skin of a rhinoceros by means of an *in vivo* antenna is difficult but possible.

The Shadwick dermis approximation leads to better simulated signal penetration through the dermis than the weighted average approximation. This slightly affects the shape of the radiation pattern due to the difference in transition of energy between the tissue layers and because the weighted average models absorb more energy. Overall, the weighted average approximation led to 0.8% more energy absorption and 1.2% greater power loss than the Shadwick approximation when considering communication between an *in vivo* antenna in the back, chest and neck and an *ex vivo* antenna on the hind leg. For the dermis, this led to 34.5% more energy being absorbed and 38.7% more power being lost than for the Shadwick approximation. When the implant is located in the chest, an *ex vivo* device on the hind leg receives 32.1% more power than when the implant is located in the neck and 217.6% more power than when the implant is located in the back. The power efficiency of the PIFA was on average 14.7% higher than the MFPEMA, although in some cases more power was received by the MFPEMA pair due to a higher input power. Based on these findings, a PIFA-based design should be implanted in the chest of the rhinoceros for optimum results.

Since the dielectric properties of rhinoceros tissue are not currently known, a novel weighted statistical model was used to approximate the permittivity and conductivity of rhinoceros organs and tissues. This multi-criteria meta-analysis of the characteristics of animals similar to the rhinoceros is unique and is believed to be the only approximation of the dielectric characteristics and evaluation of the attenuation effects for various frequencies, specifically for multiple in-vivo locations within the rhinoceros.

The weighted average models are believed to be better representatives of rhinoceros tissue since their dielectric properties were calculated at specific frequencies, whereas the frequency at which the Shadwick approximation applies is unknown. However, the observed differences in gain, electric field, power loss and specific absorption rate associated with these two approximations are quite small. Both antennas were capable of transmitting through the fat and skin of the rhinoceros models over a short distance, but communication from an *in vivo* antenna in the back, neck or chest to an *ex vivo* receiving antenna on the hind leg is not ideal for the specified antenna characteristics.

When considering communication between an *in vivo* and *ex vivo* device located on the lower back leg, our analysis indicates that a PIFA-based design should be implanted in the chest of the rhinoceros for optimum results. When considering the PIFA in the flank model, our results indicate an improvement in power efficiency of approximately 16 dbm with the introduction of a 10 mm air gap between the antenna and the agar phantom, which corresponds to the distance between the casing of the implantation device and the implanted antenna. This result demonstrates the value of the models by illustrating how effective design decisions can be made based solely on the numerical simulations when these sufficiently replicate real world conditions and characteristics. Organ and skeletal models with agar recipes to match their corresponding permittivity and conductivity values have also been designed. Although these models could not be implemented fully within the numerical model due to the resulting prohibitive complexity and increase in computational load, it is expected that they could deliver useful insights into *in vivo* propagation. This may increase the accuracy of the simulations, particularly regarding the dielectric properties and specific absorption rates of interior tissues and organs. However, this must be verified in future by means of empirical measurements.

## Conclusion

This work has described a computational model that provides a means of testing rhinoceros monitoring and tracking technology without the need for surgery and can be used to gauge the signal attenuation effects of individual constituents of rhinoceros organs and tissue. Thus, antenna and implantation designs can be optimized prior to field tests, thereby reducing the concept development period by allowing frequency dependent characteristics such as the signal penetration depth to be observed. The approximations proposed in this work are thought to be the only documented estimations of the dielectric properties of rhinoceros tissues. Therefore, the suggested agar recipes can serve as a basis for creating rhinoceros phantom tissue until such time as the dielectric properties of real rhinoceros tissue can be verified.

## Supporting information

S1 Table Physical attributes used to determine the degree of similarity between the rhinoceros and various animals. (PDF)
