# Supplementary material for: Computer simulation and physical phantom models for estimating the dielectric properties of rhinoceros tissue
Source: PLoS One. 2019 May 29;14(5):e0216595. doi: 10.1371/journal.pone.0216595 (PMC6541259; doi:10.1371/journal.pone.0216595)
Supplement: S1 Table — (PDF) [file pone.0216595.s001.pdf]

Supporting information

S1 Physical attributes used to determine the degree of similarity between the rhinoceros and various animals.

Decision Matrix for Permittivity and Conductivity Weighting Factors													
Criteria: Similarity to Rhinoceros	Available Points	Animals											
		Bovine	Canine	Equine	Feline	Frog	Human	Mouse	Ovine	Pinniped	Porcine	Rabbit	Rat
Mammal	0 – 1	1 [1]	1 [2]	1 [2]	1 [2]	0 [2]	1 [3]	1 [4]	1 [5]	1 [6]	1 [7]	1 [2]	1 [2]
Ungulate (Odd-Toed)	0 – 2	1 [8]	0*	2 [8]	0*	0*	0*	0*	1 [8]	0*	1 [8]	0*	0*
Hindgut Fermenter	0 – 1	0*	0*	1 [9]	0*	0*	0*	1 [9]	0*	0*	0*	1 [9]	1 [9]
Herbivorous	0 – 2	2 [9] [10]	1 [11] [2]	2 [9] [10]	0 [2]	1 [2]	1 [10] [12]	2 [9] [10]	2 [9] [10] [5]	1 [13]	1 [7]	2 [9] [2]	1 [2]
Primarily Solitary	0 – 1	0*	0*	0*	0*	1 [14]	0*	0*	0 [5]	0*	0 [7]	0*	0*
Territorial (Males Battle)	0 – 1	0*	1 [15]	0*	1 [15]	1 [15]	1 [15]	1 [15]	1 [5]	1 [15]	0 [7]	1 [15]	1 [15]
Horn (True Horn or Boneless)	0 – 2	1 [16] [17]	0*	0*	0*	0*	0*	0*	1 [16] [17]	0*	0*	0*	0*
Vision	0 – 2	1 [18]	1 [19] [20]	1 [18] [20]	2 [19] [20]	1 [21]	0 [18] [22]	1 [23]	1 [18]	0 [24]	1 [25]	0 [26]	1 [27]
Audition	0 – 2	1 [28] [29]	1 [28] [29]	2 [28] [29]	1 [28] [29]	0 [29]	1 [28] [29]	0 [28] [29]	1 [28] [29]	1 [30] [31]	1 [32]	0 [28] [29]	0 [29]
Olfaction	0 – 2	2 [33]	2 [34] [33]	2 [33]	1 [35]	0 [34]	1 [34]	2 [34] [33]	2 [36]	2 [37] [38]	2 [39]	1 [40] [41]	1 [34] [33]
Habitat	0 – 2	2 [42]	1 [42]	2 [42]	1 [42]	1 [42]	1*	1 [42]	1 [42]	0 [42]	1 [42]	1 [42]	1 [42]
Prehensile Lip	0 – 1	1 [43]	0*	1 [44]	0*	0*	1 [45]	0*	1 [43]	0*	0*	1 [46]	0*
Weight	0 – 2	1 [47] [48]	0 [47] [48]	1 [47] [48]	0 [49] [47] [48]	0 [49] [29]	0 [50]	0 [49] [47] [48]	0 [51] [52] [47] [48]	1 [33] [47] [47] [48]	0 [47] [48]	0 [49] [47] [48]	0 [53] [47] [48]
Skin Thickness	0 – 3	3 [54] [55]	1 [56] [57] [55]	1 [58] [55]	1 [56] [55]	0 [59]	1 [60] [?]	0 [61]	1 [1] [20]	2 [1] [20]	1 [1] [20] [55]	1 [62]	0 [60]
Collagen Content of Skin	0 – 3	3 [63] [64] [65] [66]	1 [67] [65]	3 [68] [64]	1 [69]	0 [70] [71]	2 [65] [66] [60]	1 [72]	2 [64] [66]	3 [73]	3 [63] [64] [65] [66] [74]	1 [75]	1 [65] [53]
Fat Layer	0 – 3	2 [76] [77]	0 [57]	2 [78] [79]	0 [80]	0*	1 [81] [82]	0 [83]	1 [51] [84] [52] [77]	2 [85] [1] [20]	2 [77] [20]	0 [86]	0 [87]
Body Length	0 – 2	2 [88] [12]	0 [89]	3 [90] [12]	0 [49]	0 [49]	1 [49]	0 [31]	1 [91]	2 [33]	1 [92]	0 [49]	0 [93]
Body Height	0 – 2	2 [88] [12]	1 [89] [29]	2 [29] [12]	0 [29]	0 [29]	2 [50]	0 [94]	1 [32]	2 [33]	1 [34]	0 [29]	0 [29]
Intestines/Organs	0 – 3	2 [95] [12]	0 [96] [97]	3 [98] [12]	0 [96] [97]	0 [99]	1 [96] [100]	0 [101]	1 [96] [102]	1 [103]	1 [96] [102]	0 [96]	0 [96] [101]
Skeletal Structure	0 – 3	3 [104] [60] [12]	1 [57] [97]	2 [98] [12]	1 [104] [97]	0 [104] [99]	1 [96] [100]	1 [105]	1 [96] [106]	1 [103]	2 [104]	1 [107]	1 [96]
Straight Legs	0 – 1	1 [95] [12]	1 [57] [97]	1 [98] [12]	1 [104] [97]	0 [99]	1 [100]	0 [105]	1 [106]	0 [103]	1 [104]	0 [107]	0 [107]
Swiveling Ears	0 – 1	1 [95]	1 [108]	1 [109]	1 [108]	0 [110]	0 [100]	1 [111]	1 [112]	0 [103]	0 [113]	1 [114]	1 [115]
Thermal Properties of Organs	0 – 3	3 [96] [116] [117]	1 [96] [117]	3 [118]	1 [96]	0 [119]	2 [96] [117]	0 [120]	2 [96] [116] [117]	2 [96] [117] [118]	2 [96] [116] [117]	1 [96] [117]	1 [96]
Water Content of Organs	0 – 3	2 [77] [118]	1 [121]	3 [122] [118]	2 [123]	0 [124]	3 [125] [121]	1 [126]	2 [52] [77] [118]	2 [127] [118]	2 [77] [118]	1 [128]	1 [128]
Points Total	Max (48)	37	16	39	15	5	22	13	26	24	24	14	12
Multiplier Total	Max (327)	262	85	264	84	11	151	67	164	174	171	89	69

* Indicates behavioural or physical attributes that are not distinctly profound or directly comparable to the rhinoceros since their function or usage may vary depending on the breed, location or species. It also indicates commonly known animal traits that are consequently not supported by a citation.

References

1. National Geographic.
   "Animals",
   [Online]. Available: http://www.nationalgeographic.com/animals. [Accessed 3 June 2016].
   2016.

2. Baxland B.
   "Humans are Mammals",
   Australian Museum.
   [Online]. Available: https://australianmuseum.net.au. [Accessed 12 October 2015].
   2015.

3. New World Encyclopedia.
   "Mouse",
   [Online]. Available: http://www.newworldencyclopedia.org/entry/Mouse. [Accessed 2 February 2017].
   2008.

4. New World Encyclopedia.
   "Sheep",
   [Online]. Available: http://www.newworldencyclopedia.org/entry/Sheep. [Accessed 2 February 2017].
   2008.

5. National Geographic.
   "California Sea lion",
   [Online]. Available: http://www.nationalgeographic.com/animals/mammals/c/california-sea-lion/. [Accessed 26 May 2016].
   2016.

6. New World Encyclopedia.
   "Pig",
   [Online]. Available: http://www.newworldencyclopedia.org/entry/Pig. [Accessed 2 February 2017].
   2008.

7. Huffman B.
   "Ungulates of the World",
   [Online]. Available: Ultimateungulate.com. [Accessed 16 February 2016].
   2014.

8. The Open University.
   "Studying Mammals: Plant Predators",
   [Online]. Available: https://www.open.edu/openlearn. [Accessed 16 March

2016].
2016.

9. Mills M.
"The Comparative Anatomy of Eating",
[Online]. Available: ecologos.org. [Accessed 12 Febraury 2016].
1996.

10. Patry K.
"Carnivore Digestive System",
[Online]. Available: raising-rabbits.com. [Accessed 12 Febraury 2016].
2016.

11. McArdle J.
"Humans are Omnivores",
Vegetarian Journal.
[Online]. Available: https://www.vrg.org/nutshell/omni.htm. [Accessed 13 Febraury 2016].
1991.

12. New World Encyclopedia.
"Seal",
[Online]. Available: http://www.newworldencyclopedia.org/entry/Seal. [Accessed 2 February 2017].
2008.

13. Messenger S.
"21 Animals Who Know Being Single Is Awesome",
[Online]. Available: https://www.thedodo.com/. [Accessed 2 February 2017].
2015.

14. BBC.
"Territorial",
[Online]. Available: http://www.bbc.co.uk/nature/adaptations/Territory_(animal). [Accessed 27 May 2016].
2014.

15. Myers P, Espinosa R, Parr C, Jones T, Hammond G, Dewey T.
"Horns and Antlers",
[Online]. Available: https://animaldiversity.org/collections/mammal_anatomy/horns_and_antlers/. [Accessed 27 May 2016].
2016.

16. Culp K.
"Antlers vs. Horns",

Fossil Rim Wildlife Center.
[Online]. Available: https://fossilrim.org/2014/04/02/antlers-vs-horns/.
[Accessed 25 May 2016].
2014.

17. All About Eyes.
"The Best Eyes In The Animal Kingdom",
[online]. Available at: https://allabouteyes.com/best-eyes-animal-kingdom.
[Accessed 27 May 2016].
2016.

18. Morgans Lists.
"10 Examples of How Animals See - Images That Show Us The World Through Their Eyes",
[online]. Available at: http://morgana249.blogspot.com/2014/07/10-examples-of-how-animals-see-images.html. [Accessed 27 May 2016].
2014.

19. Web Ecoist.
"How Do They See? Views Through the Eyes of 7 Animals".
[online]. Available at:
https://www.momtastic.com/webecoist/2009/01/14/animal-vision-color-detection-and-color-blindness/. [Accessed 26 May 2016].
2017.

20. Khanwilkar S.
"'Eye' Wonder: 10 Things You Didn't Know About Frog Eyes",
[online]. Available at: https://jhwildlifefilm.wordpress.com/2014/07/28/eye-wonder-10-things-you-didnt-know-about-frog-eyes/. [Accessed 26 May 2016].
2014.

21. Museum of Vision.
"Animal Eyes",
The Foundation of the American Academy of Ophthalmology.
[online]. Available at:
http://www.kyeyemds.org/Resources/Documents/Animal%20Eyes%20(2).
[Accessed 27 May 2016].
1997.

22. Baker M.
"Neuroscience: Through the eyes of a mouse",
Nature, vol. 502, pp. 156-158, 2013.

23. Hanke F, Hanke W, Scholtyssek C, Dehnhardt G.
"Basic mechanisms in pinniped vision",
Experimental Brain Research, vol. 199, pp. 299-311, 2009.

24. Velarde A, Llonch P, Dalmau A.
“Pig vision and management/handling”,
[online]. Available at:
https://www.pig333.com/articles/pig-vision-and-management-handling_981/.
[Accessed 27 May 2016].
2009.

25. Random Rabbit Pages.
“A Rabbit’s Vision”,
[Online]. Available: http://www.vgr1.com/vision/. [Accessed 6 February 2017].
2017.

26. Rat Sensory World.
“What Do Rats See?”,
[Online]. Available: http://www.ratbehavior.org/RatVision.htm. [Accessed 6 February 2017].
2004.

27. Ageuk.
“How does an animal’s hearing compare to a human’s – Infographic”,
[Online]. Available: http://www.ageukhearingaids.co.uk/hearing-aid-news/how-does-animals-hearing-compare-humans-infographic. [Accessed 6 February 2017].
2016.

28. Louisiana State University.
“How Well Do Dogs and Other Animals Hear?”,
[Online]. Available: http://www.lsu.edu/deafness/HearingRange.html.
[Accessed 6 February 2017].
2003.

29. Reichmuth C, Holt M, Mulsow J, Sills J, Southall B.
“Comparative assessment of amphibious hearing in pinnipeds”.
Journal of Comparative Physiology, vol. 199, no. 6, pp. 491-507, 2013.

30. Hemila S, Nummela S, Berta A, Reuter T.
“High-frequency hearing in phocid and otariid pinnipeds: An interpretation based on inertial and cochlear constraints (L)”.
The Journal of the Acoustical Society of America, vol. 120, no. 6, pp. 3463-3466, 2006.

31. Kittawornrat A, Zimmerman J.
“Toward a better understanding of pig behavior and pig welfare”.
Animal Health Research Reviews, vol. 12, no. 1, pp. 25-32, 2010.

32. Viegas J.
“10 Best Sniffers in the Animal Kingdom”.
[Online]. Available: https://www.seeker.com/10-best-sniffers-in-the-animal-kingdom-1768828254.html. [Accessed 6 February 2017].

2014.

33. Ache B, Young J.
"Olfaction: Diverse Species, Conserved Principles",
Neuron, vol. 48, no. 3, pp. 417-430, 2005.

34. Wexler-Mitchell E.
"Smell - a Very Important Sense to Cats".
[Online]. Available: https://catcare.com. [Accessed 7 February 2017].
2010.

35. Dwyer C.
"The Welfare of Sheep".
Springer Science & Business Media, New York, NY, United States, p. 136, 2008.

36. Pitcher B, Harcourt R, Schaal B, Charrier I.
"Social Olfaction in Marine Mammals: Wild Female Australian Sea Lions can Identify Their Pup's Scent".
Biology Letters, vol. 7, no. 1, pp. 60-62, 2010.

37. Van Valkenburgh B, Curtis A, Samuels J, Bird D, Fulkerson B, Meachen-Samuels J, Slater G.
"Aquatic adaptations in the nose of carnivorans: evidence from the turbinates".
Journal of Anatomy, vol. 218, no. 3, pp. 298-310, 2011.

38. Brunjes P, Feldman S, Osterberg S.
"The Pug Olfactory Brain: A Primer".
Chemical Senses, vol. 41, no. 5, pp. 415-425, 2016.

39. Le Gros Clark W.
"Observations on the Structure and Organization of Olfactory Receptors in the Rabbit".
The Yale Journal of Biology and Medicine Inc, vol. 29, no. 2, pp. 83-95, 1956.

40. Xi J, Si X, Kim J, Zhang Y, Jacob R, Kabilan S, Corley R.
"Anatomical Details of the Rabbit Nasal Passages and Their Implications in Breathing, Air Conditioning, and Olfaction".
The Anatomical Record, vol. 299, no. 7, pp. 853-868, 2016.

41. Enchanted Learning.
"Biomes - Habitats",
[Online]. Available:
http://www.enchantedlearning.com/biomes/desert/desert.shtml. [Accessed 6 February 2017].
2016.

42. Parrington C.
"The British Cyclopaedia of Natural History: Combining a Scientific Classification of Animals, Plants, and Minerals".
Orr & Smith, London, vol. 3, p 171, 1837.

43. Mills D, McDonnel S, eds.
"The Domestic Horse: The Origins, Development and Management of Its Behaviour",
Cambridge University Press, Cambridge, United Kingdom, p. 100, 2005.

44. Allan K, eds.
"The Routledge Handbook of Linguistics",
Routledge, p. 28, 2015.

45. House Rabbit Society.
"Common Illnesses",
[Online]. Available: https://www.hrss.net/aar/health/health_dental.html.
[Accessed 5 February 2017].
2003.

46. The Website of Everything.
"Mammals Ordered by Weight",
[Online]. Available: https://thewebsiteofeverything.com. [Accessed 5 February 2017].
2011.

47. Warrignton P.
"Animal Weights and Their Food and Water Requirements",
Government of British Columbia, 2001.

48. Kinsere P.
"What is the relationship between brain and body size?",
Serendip Studio.
[Online]. Available: https://serendipstudio.org/exchange/brains/compare/size5.
[Accessed 2 February 2017].
2012.

49. New World Encyclopedia.
"Human being",
[Online]. Available: http://www.newworldencyclopedia.org/entry/Human_being.
[Accessed 2 February 2017].
2009.

50. O'Rourke B, Russel R, UW-Madison D.
"Lamb Carcass Evaluation",
[Online]. Available:
sheboygan.uwex.edu/files/2010/08/Lamb-Carcass-Evaluation.pdf. [Accessed 26

May 2016].
2010.

51. Aganga A.
“Water utilization by sheep and goats in northern Nigeria”,
Food and Agriculture Organization of the United Nations, 1986.

52. Smith Q.
“Body weight, cutaneous collagen and hexosamine of cortisone-treated female rats of various ages”,
The Journal of Investigative Dermatology, vol. 42, no. 5, pp. 353-357, 1964.

53. Asante-Poku A, Aning, K, Boi-Kikimoto B, Yeboah-Manu D.
“Prevalence of bovine tuberculosis in a dairy cattle farm”,
The Onderstepoort Journal of Veterinary Research, vol. 81, no. 2, p. 6, 2014.

54. Jarolim E.J.
“Comparative Medicine: Anatomy and Physiology”,
Springer Science & Business Media, New York, p. 111, 2014.

55. Cline J.
“Skin and Coat”,
[Online]. Available:
https://cdn.shopify.com/s/files/1/0659/4529/files/SkinCoat_WhiteSheet6-5.pdf?6678352109863082785. [Accessed 2 February 2017].
2017.

56. Evans H, de Lahunta A.
”Miller’s Anatomy of the Dog”,
Elsevier Health Sciences, New York, p. 62, 2013.

57. Volkering M.
“Variation of skin thickness over the equine body and the correlation between skin fold measurement and actual skin thickness”,
Faculty of Veterinary Medicine, Utrecht University, Netherlands, 2009.

58. Kaplan M, Heimes P, Zarza E, McCormack J.
“On the morphology of Plectrohyla chryses (Anura: Hylida; Hylini), with comments on some controversial characters, phylogenetic relationships, and diagnosis of this species”,
Caldasia, vol. 38, no. 2, pp. 257-273, 2016.

59. Meyers M, Chen P, Lin A, Seki Y.
“Biological Materials: Structure and Mechanical Properties”,
Progress in Materials Science, vol. 53, no. 1, pp. 1-206, 2008.

60. Manne J, Markova M, Siracusa L, Jimenez S.
"Collagen Content in Skin and Internal Organs of the Tight Skin Mouse: An Animal Model of Scleroderma",
Biochemistry Research International, vol. 2013, no. 1, p. 8, 2013.

61. Tao Y.
"Studies on the Quality of Rex Rabbit Fur",
World Rabbit Science, vol. 2, no. 1, pp. 21-24, 1994.

62. Pearson A, Dutson T.
"Inedible Meat by-Products",
Springer Science & Business Media, vol. 8, p. 226, 2013.

63. Food and Agriculture Organization of the United Nations.
"Definition and Classification of Commodities",
[Online]. Available: http://www.fao.org/ES/faodef/fdef19e.htm#19.1.
[Accessed 26 May 2016].
1994.

64. Neuman R, Logan M.
"The Determination of Collagen and Elastin in Tissues".
Journal of Biological Chemistry, vol 186, no. 2, pp 549-556, 1950.

65. Ovinex.
"Premium Sheep Collagen",
[Online]. Available: http://ovinex.com/collagen.php. [Accessed 25 May 2016].
2010.

66. Andersen ML, Winter LMF.
"Animal Models for Biomedical Research",
Institute of Laboratory Animal Resources and the American College of Laboratory Animal Medicine, Washington D.C., USA, vol. 3, p. 83, 1970.

67. De Souza M, Silva M, de Oliveira Pinto J, de Souza Lima M, Crepaldi J, Lopes G, Dos Santos H, de Azambujo Ribeiro R, Thome R.
"Immunohistochemical Expression of Collagens in the Skin of Horses Treated with Leukocyte-Poor Platelet-Rich Plasma",
Biomed Research International, 2015.

68. Dokuzeylul B, Altun E, Ozdogan T, Bozkurt H, Arun S, Or M.
"Cutaneous asthenia (Ehlers–Danlos syndrome) in a cat",
Turkish Journal of Veterinary and Animal Sciences, vol. 37, pp. 245-249, 2013.

69. Sai K, Babu M.
"Studies on Rana tigerina skin collagen",
Comparative Biochemistry and Physiology Part B, vol. 128, no. 1, pp. 81-90, 2000.

70. Li H, Liu B, Gao L, Chen H.
"Studies on bullfrog skin collagen",
Food Chemistry, vol. 84, no. 1, pp. 65-69, 2003.

71. Long K, Artlett C, Blankenhorn E.
"Tight skin 2 mice exhibit a novel time line of events leading to increased extracellular matrix deposition and dermal fibrosis",
Matrix Biology, vol. 38, pp. 91-100, 2014.

72. Khamas W, Smodlaka H, Leach-Robinson J, Palmer L.
"Skin histology and its role in heat dissipation in three pinniped species",
Acta Veterinaria Scandinavica, vol. 54, no. 1, no. 46, 2012.

73. Rodrigues F, Martins V, Plepis A.
"Porcine Skin as a Source of Biodegradable Matrices: Alkaline Treatment and Glutaraldehyde Crosslinking",
Polimeros, vol. 20, no. 2, pp. 92-97, 2010.

74. Nimni M, de Guia E, Bavetta L.
"Collagen, Hexosamine and Tensile Strength pf Rabbit Skin During Aging",
The Journal of Investigative Dermatology, vol. 47, no. 2, pp. 156-158, 1966.

75. Johnson E, Vidyadaran M.
"An Evaluation of Different Sites for Measuring Fat Thickness in the Beef Carcass to Determine Carcass Fatness",
Australian Journal for Agricultural Research, vol. 32, no. 6, pp. 999-1007, 1981.

76. Food and Agriculture Organization of the United Nations.
"Meat, Fat and Other Edible Carcass Parts",
Food and Agriculture Organization of the United Nations.
2010.

77. Superchi P, Vecchi I, Beretti V, Sabbioni A.
"Relationship among BCS and Fat Thickness in Horses of Different Breed, Gender and Age",
Annual Research & Review in Biology, vol. 4, no. 2, pp. 354-365, 2013.

78. Kentucky Equine Research.
"Rump Fat Measurement in Horses",
[Online]. Available:
http://saracen.equinews.com//article/rump-fat-measurement-horses. [Accessed 26 May 2016].
2015.

79. Matton J, Nyland T.
"Small Animal Diagnostic Ultrasound",
Elsevier Health Sciences, pp. 470-472, 2014.

80. Ho D, Kim E.
"Optical Skin-fat Thickness Measurement Using Miniaturized Chip LEDs: A Preliminary Human Study",
Journal of the Optical Society of Korea, vol. 13, no. 3, pp. 304-309, 2009.

81. Ng J, Rohling R, Lawrence PD..
"Automatic Measurement of Human Subcutaneous Fat with Ultrasound".
IEEE Transactions on Ultrasonics, Feroelectrics and Frequency Control, vol. 56, no. 8, pp. 1642-1653, 2009.

82. Poligone B, Hayden M, Chen L, Pentland A, Jimi E, Ghosh S.
"A Role for NF-KB Activity in Skin Hyperplasia and the Development of Keratoacanthomata in Mice",
PLoS ONE, vol. 8, no. 8, 2013.

83. Bruwe G, Naude R.
"An evaluation of the lamb and mutton carcase grading system in the Republic of South Africa. 1. A survey of carcase characteristics of the different grades",
South African Journal of Animal Science, vol. 17, no. 2, pp. 79-84, 1987.

84. Trites A, Jonker R.
"Morphometric measurements and body condition of healthy and starveling Steller sea lion pups (Eumetopias jubatus)",
Aquatic Mammals, vol. 26, no. 2, pp. 151-157, 2000.

85. Pascual J, Blanco J, Piquer O, Quevedo F, Cervara C.
"Ultrasound Measurements of Perirenal Fat Thickness to Estimate the Body Condition of Reproducing Rabbit Does in Different Physiological States",
World Rabbit Science, vol. 12, no. 1, pp. 7-21, 2012.

86. Berlanga-Acosta J, Vázquez-Blomquist D, Cibrián D, Mendoza Y, Ochagavia M, Miranda J, Suarez J, Gonzalez-Ferrer Y, Vila J, Abreu A, Ugarte-Moreno D, Cruz Y, Howland I, Coro-Antich R, Leon O, Bringas R, Barco D.
"Growth Hormone Releasing Peptide 6 (GHRP6) reduces liver fibrosis in CCl4 chronically intoxicated rats",
Biotechnologia Aplicada, vol. 29, no. 2, pp. 60-72, 2012.

87. Bene S, Nagy B, Nagy L, Kiss B, Polgar J, Szabo F.
"Comparison of body measurements of beef cows of different breeds",
Archives Animal Breeding, vol. 50, no. 4, pp. 363-373, 2007.

88. My Pet.
"How to Measure your Dog (for Coats, Hats, Jumpers & Dog Clothes)",
[Online]. Available: http://www.mypet.net.au/sizeguide.htm. [Accessed 26 May 2016].
2008.

89. Federal Highway Administration.
"Equestrian Design Guidebook for Trails, Trailheads and Campgrounds".
[Online]. Available:
https://www.fhwa.dot.gov/environment/recreational_trails/publications/fs_publications/07232816/.
[Accessed 28 May 2016].
2014.

90. Magged M, Bernere D, Julke H, Hohaus C, Brehm W, Gerlach K.
"Is sheep lumbar spine a suitable alternative model for human spinal researches? Morphometrical comparison study",
Laboratory Animal Research, vol. 29, no. 4, pp. 183-189, 2013.

91. Ramesh S, Sivakumar T, Gnanaraj T, Murallidharan R, Murugan M.
"Comparative Performance of Landrace and Large White Yorkshire Pigs Under Tropical Maritime Monsoon Climate",
Turkish Journal of Veterinary and Animal Sciences, vol. 40, no. 1, pp. 42-46, 2009.

92. The New World Encyclopedia.
"Rat",
[Online]. Available: http://www.newworldencyclopedia.org/entry/Rat.
[Accessed 4 April 2017].
2008.

93. Alterman L, Doyle G, Izard M.
"Creatures of the Dark",
Springer Science and Business Media, p. 553, 2013.

94. Budras KD, Habel RE, Mulling CK, Greenough PR, Jahrmarker G, Richter R, Starke D.
"Bovine Anatomy: An Illustrated Text",
Schluterse, Berlin, pp. 68-69, 2003.

95. Holmes K.
"Thermal Properties",
[Online]. Available: users.ece.utexas.edu/ valvano/research/Thermal.pdf.
[Accessed 25 February 2016].
2009.

96. Jack C, Watson P.
"Veterinary Technician's Daily Reference Guide: Canine and Feline",
John Wiley & Sons, New Jersey, USA, 2011.

97. Parker R.
"Equine Science",
Cengage Learning, Hampshire, UK, pp. 88-97, 2012.

98. Wingerd B.
"Frog Dissection Manual,
JHU Press, Baltimore, USA, pp. 18-19, 1998.

99. Saladin K.
"Human Anatomy",
McGraw Hill Higher Education, New York, USA, pp. 24-43, 2007.

100. Albers T, Pritchett-Corning K .
"Diagnostic Necropsy and Selected Tissue and Sample Collection in Rats and Mice",
Journal of Visualized Experiments, vol. 54, no. 54, pp. 2966, 2011.

101. Iaizzo P.
"Handbook of Cardiac Anatomy, Physiology, and Devices",
Springer Science & Business Media, Berlin, Germany, pp. 102-103, 2009.

102. Perrin W, Wursig, B, Thewissen J.
"Encyclopedia of Marine Mammals",
Academic Press, Cape Town, South Africa, pp. 31-33, 2009.

103. Oxford University Museum of Natural History.
"Animal Skeletons",
[Online]. Available: https://www.oumnh.ox.ac.uk/. [Accessed 25 February 2016].
2005.

104. Bab I, Hajbi-Yonissi C, Gabet Y, Muller R.
"Micro-Tomographic Atlas of the Mouse Skeleton",
Springer Science & Business Media, Berlin, Germany, pp. 149-163, 2007.

105. Aus-Meat.
"Handbook of Australian Meat".
[Online]. Available: https://catalogue.nla.gov.au/Record/299475. [Accessed 25 February 2016].
2006.

106. Fraser M, Girling S.
"Rabbit Medicine and Surgery for Veterinary Nurses",
John Wiley & Sons, New York, USA, pp. 106-114, 2009.

107. Slatter D.
"Textbook of Small Animal Surgery",
Elsevier Health Sciences, Amsterdam, Netherlands, vol. 1, pp. 1736-1768, 2003.

108. Budras K, Sack W, Rock S.
"Anatomy of the Horse: An Illustrated Text".

Schlutersche, Berlin, 2003.

109. van Dijk P, Mason M, Schoffelen R, Narins P, Meenderink S.
"Mechanics of the frog ear",
Hearing Research, vol. 273, no. 1-2, pp. 46-58, 2011.

110. Cox T, Camci E, Luquetti D, Turner E.
"The genetics of auricular development and malformation: New findings in model systems driving future directions",
European Journal of Medical Genetics, vol. 57, no. 8, pp. 394-401, 2014.

111. Seibel V, Lavinsky L, Irion K.
"CT-Scan sheep and human inner ear morphometric comparison",
Brazilian Journal of Otolaryngology, vol. 72, no. 3, pp. 370-376, 2006.

112. Veterian Key.
"Porcine Clinical Procedures".
[Online]. Available: https://veteriankey.com/porcine-clinical-procedures/.
[Accessed 24 February 2016].
2016.

113. Rickel J.
"Rabbit Ears: A Structural Look: injury or disease, can send your rabbit into a spin".
House Rabbit Society.
[Online]. Available: https://rabbit.org/rabbit-ears-a-structural-look-2/.
[Accessed 12 February 2016].
2012.

114. Duocomm D.
"Rats: Practical, Accurate Advice from the Expert".
i5 Publishing, California, USA, 2011.

115. Bhattacharya A, Mahajan R.
"Temperature dependence of thermal conductivity of biological tissues",
Physiological Measurement, vol. 24, no. 3, 2003.

116. Choi J, Bishof J.
"Review of biomaterial thermal property measurements in the cryogenic regime and their use for prediction of equilibrium and non-equilibrium freezing applications in cryobiology",
Cryobiology, vol. 60, no. 1, pp. 52-70, 2009.

117. American Society of Heating, Refrigeration and Air-conditioning Engineers.
"Thermal Properties of Foods". In: "2006 ASHRAE handbook: refrigeration",
ASHRAE, Atlanta, USA, 2006.

118. Hart F, Dunfee W.
"In vivo measurement of the low-frequency dielectric spectra of frog skeletal muscle",
Physics in Medicine and Biology, vol. 38, no. 8, pp. 1099-1112, 1993.

119. Lubner S, Choi J, Hasegawa Y, Fong A, Bishof J, Dames C.
"Measurements of the Thermal Conductivity of Sub-Millimeter Biological Tissues",
International Mechanical Engineering Congress & Exposition (IMECE), Houston, Texas, USA, vol. 7, pp. 1397-1404, 2012.

120. Maksymowicz K, Marycz K, Szotek S, Kalinsko K, Serwa E, Lukomski E, Czogala J.
"Chemical composition of human and canine fascia lata",
Acta Biochimica Polonica (ABP), vol. 59, no. 4, pp. 531-535, 2012.

121. Marshall D.
"Horse Health Depends on Water".
University of Delaware, College of Agriculture and Natural Resources.
[Online]. Available:
https://4-h.org/wp-content/uploads/2016/02/WaterSources.pdf. [Accessed 21 May 2016].
2004.

122. Syufry F.
"How Much Water Does an Adult Cat Need to Drink",
[Online]. Available: http://cats.about.com/od/waterforcats/f/waterneeds.htm.
[Accessed 26 May 2016].
2016.

123. Behler J, Behler D.
"Frogs: A Chorus of Colors",
Sterling Publishing Company, New York, USA, p. 54, 2008.

124. The USGS Water Science School.
"The water in you".
[Online]. Available: https://water.usgs.gov/edu/propertyyou.html. [Accessed 2 May 2016].
2016.

125. Fox J, Barthold S, Davisson M, Newcomer C, Quimby F, Smith A.
"The Mouse in Biomedical Research: Normative Biology, Husbandry and Models",
Elsevier, Amsterdam, Netherlands, vol. 3, p. 76, 2006.

126. Le Boeuf B, Laws R.
"Elephant Seals: Population Ecology, Behavior, and Physiology",

University of Califronia Press, California, USA, pp. 103-105, 1994.

127. Mayer W.
"Physiological Mammalogy",
Elsevier, Amsterdam, Netherlands, vol. 2, p. 132, 2012.

128. Huffman B.
"Cow Mammal",
Encyclopedia Britannica.
[Online]. Available: https://www.britannica.com/animal/cow. [Accessed 11 November 2016].
2016.
